# Perception, practices, and understanding related to teenage pregnancy among the adolescent girls in India: a scoping review

**DOI:** 10.1186/s12978-023-01634-8

**Published:** 2023-06-21

**Authors:** Arpita Panda, Jayashree Parida, Susangita Jena, Abinash Pradhan, Sanghamitra Pati, Harpreet Kaur, Subhendu Kumar Acharya

**Affiliations:** 1 ICMR-Regional Medical Research Center, NALCO Nagar, Chandrasekharpur, Bhubaneswar, Odisha 751023 India; 2 Division of Epidemiology and Communicable Diseases (ECD-Tribal Health), ICMR Head Quarters, New Delhi, India

**Keywords:** Teenager, Pregnancy, Adolescent girls, India, Perceptions, Practices, Contraceptive use, Abortion

## Abstract

**Background:**

Teenage pregnancy is a concerning public health problem in India. Misperception and misunderstanding about pregnancy and its preventive methods lead to pregnancy when adolescents are involved in unsafe sexual intercourse. This scoping review aims to discuss the evidence on the perception, practices, and understanding related to teenage pregnancy among adolescent girls in the Indian context.

**Method:**

The Arksey and O'Malley scoping review framework and Joanna Briggs Institute Reviewers’ Manual were used for the scoping review. The Population, Concept, and Context strategy (PCC) ensured the review questions, eligibility criteria, and search strategy. The Systematic Review and Meta-analysis: Extension for Scoping Review (PRISMA-ScR) was used. A literature search was done using electronic databases by specific keywords such as “teenage”, “adolescences”, “pregnancy”, “perception”, “knowledge”, “awareness”, etc. Relevant grey literature was identified through further searching. The review included studies that fulfil inclusion criteria having female adolescent groups aged from 10 to 19 years in the Indian context between the years 2000 and 2021.

**Results:**

We found 40 eligible studies; more than half of these were from southern (35%) and northern (27.5%) regions, and studies from the rest of India were very sporadically distributed. Most studies (72.5%) were published in the last 10 years. The relevant extracted data from individual studies were synthesized and presented in the two major sections, perception, practices, and the second one, understanding and experiences among teenage girls. The understanding of pregnancy and teenage pregnancy-related preventive methods was detailed analysis in about 72% of papers whereas other aspects, such as perception (22.5%), practices (25%), and experiences (7.5%) were discussed in the remaining papers related to pregnancy among adolescent girls.

**Conclusion:**

Evidence in the selected studies shows that understanding and practices are the major areas that were primarily explored, where perception, practices and experiences are the topics that are relatively less investigated. Literature synthesis derives misconception, lack of understanding, and practices without knowing the consequences are the key factors responsible for early pregnancies. Future interventions like increasing awareness, providing comprehensive reproductive knowledge, convenient health care aids, and proper counselling are adequate measures for minimalising the problem. The present analysis showed that studies are limited in their scope concerning various aspects of teenage pregnancy in India, so this scoping review gives essential perspectives on future research and implementation plans and policies in this field.

**Supplementary Information:**

The online version contains supplementary material available at 10.1186/s12978-023-01634-8.

## Background

The United Nations Children Fund (UNICEF) defines “teenage pregnancy” as when an adolescent girl becomes pregnant between the age group 13–19 years [1]. Pregnancy in adolescents is a risk event because the body of an adolescent is still growing and not entirely prepared for reproduction [2]. Approximately 20,000 girls give birth before 18 years, which is 7.3 million births annually. Of them, 95% of childbirths among adolescent girls occur in low and middle-income countries [3] and at least 777,000 births occur among teenage girls below 15 years [4]. According to the National Family Health Survey-4 (2015–16), adolescent childbearing in India is a significant health concern [5].

Adolescence is the crucial period of life where a child passes through sexual and psychological maturity [6]. The onset of puberty with sexual maturity makes them curious about exploring their body, resulting in risky sexual behaviours [7]. Studies report that early sexual initiation increases adolescents’ risk of unwanted pregnancy [8, 9]. Evidence also indicates that unsafe sexual practices with misperceptions and misunderstandings about sexuality, conception, reproductive health, pregnancy, and contraception methods are the leading causes of teenage pregnancy [10–13]. Adolescent pregnancy is associated with several medical complications, such as anemia, preterm and post-term complications, preeclampsia, low birth weight, pregnancy-induced hypertension, and miscarriage [14–16]. Several previous studies have also confirmed that though the experiences on social consequences of teenage pregnancy among married and unmarried adolescents differ, the leading underlying causes are the same: lack of understanding and unsafe sexual practices [17–19]. Unwanted pregnancy before marriage often leads to risky childbirth and increased lifetime risk for the pregnant or delivering adolescent. The primary reasons are a lack of correct understanding of abortion, various attached socio-cultural stigmas, lack of proper counselling services, lack of provision for contraceptives, and the nonavailability of adequate health care. Available literature indicates that unintended teenage pregnancies result in self-induced or unsafe abortion by untrained or nonprofessional personnel/services [20, 21]. Evidence from India shows that among 2 million adolescent pregnancies in the country annually, more than half of cases end up with abortion, and more than three-fourths of abortions are unsafe [22]. Available literature shows that due to a lack of understanding about abortion, unsafe pregnancy termination results in hemorrhage, sepsis, and trauma, the 4th leading cause of maternal mortality [23].

Several of the conservative and regressive characteristics of Indian society significantly affect the perceptions, practices, and understanding related to adolescent or teen pregnancy among girls in India. In this context, while talking ‘perception’ around teen pregnancy, it corresponds to how people including teens and others in their around, interpret and comprehend the occurrence of teen pregnancy. Perception in such cases encompasses people's views and ideas about adolescent motherhood as well as their ideas of the causes, effects, and ramifications of teen pregnancy. ‘Practices’ in the context of teen pregnancy generally refer to the attitudes, acts, and routines that teens follow around sexual health, contraception, and preventing pregnancy. Indian societal framework is mostly conservative, patriarchal, and highly value-oriented, so premarital sex and particularly regarding girls, is considered highly immoral, bringing severe social consequences. Therefore, discussions, knowledge, and information about sex often leads to humiliation including in schools, as it is regarded a taboo [24]. In a scenario as discussed above, even the conversation about sexual health among adolescents happens only through whispering in most cases [25]. So, it requires a detail analysis of the availability of evidence and the scope for further research in this aspect. Furthermore, due to inadequate knowledge among them, parents, peers, and adults in most cases fail to give necessary guidance and information on good and safe practices regarding sexual behaviour to children [26]. In such restrictive scenario, ‘understanding of teen pregnancy’ associated with confusions is causing a significant gap in knowledge and information access among these adolescents. In some cases, the adolescent girls, to satisfy their curiosity, end up getting information from social media and same-age peer groups, resulting in incomplete understanding and perceptions, misinformation, and harmful practices [27]. Furthermore, various social agencies, religious bodies, and sociocultural institutions also play pivotal roles by pushing further restrictions like the ban on associated aid such as emergency contraceptives, etc [28]. In such scenario, explaining the availability of sufficient evidence around various aspects of adolescents’ engagement in sexual activity is important. Similarly, understanding research trend and pattern addressing and investigating teen perception, practices, and understanding are crucial steps in mitigating the risks and issue of teenage pregnancy in Indian context.

In this context, the present scoping review encompassing the various evidence and literature available on this topic aims to provide crucial insights for advancing research on sexual risk behaviours among adolescents. The pattern and trend of research around adolescents’ perceptions about sexual contact, pregnancy, early childbearing and their understanding regarding conception, contraception, family planning, and abortion that is reflected in their practices, requires clear and in-depth research in the Indian context. So, by exploring such aspects, the present scoping review will also improve future direction of studies on the Adolescent Sexual and Reproductive Health (ASRH) programme. To our knowledge, no scoping review was undertaken in India related to the perceptions and practices among adolescent girls and their experience and understanding of pregnancy. The aim of this scoping review is to synthesize evidence concerning teen/adolescent pregnancy and discuss the gaps in perceptions, practices, and understanding of teen pregnancy among Indian adolescent girls.

## Objectives

The present review has two major objectives, first, map the evidence around the perceptions and practices related to early/teenage pregnancy among Indian adolescent girls and in second, studying the status of the available literature on experiences and understanding related to early pregnancy among married and unmarried adolescents with past pregnancy experiences in India.

## Methods

The present scoping review followed the Preferred Reporting Items for Systematic Reviews and Meta-Analyses Extension for Scoping Reviews (PRISMA-ScR) guidelines to report the evidence. Following five-stage methodological framework was used according to Arksey and O'Malley’s scoping review framework (2005) and the Joanna Briggs Institute Reviewers’ Manual [29]: (1) identifying the research question and (2) the relevant studies; (3) selecting the studies according to inclusion criteria; (4) charting and interpreting data; and (5) summarizing and reporting of results [30].

Identifying the research questions: The research questions of this scoping review on perception, practices and understanding related to teenage pregnancy among adolescent girls are:

- What is the status of evidence available around the perceptions and practices related to early/teenage pregnancy among Indian adolescent girls?
- What is the status of the available literature on experiences and understanding related to early pregnancy among married and unmarried adolescents with past pregnancy experiences in India?
- What is the scope for scaling up of evidence around teen pregnancy in India?

Identifying the relevant studies:

Information source and search strategy: A comprehensive literature search was conducted by the two reviewers (AP and SJ) to identify all relevant studies in different e-databases such as EMBASE, SCOPUS, Web of Science, PubMed, PsycINFO, MeSH terms "teenage"; "adolescence"; "young youth"; "pregnancy"; "perception"; "knowledge"; "awareness"; "married"; "unmarried"; "mother"; "abortion"; "India" were used for the literature search (Additional file 1), etc. Also we searched Google scholar and Researchgate for gray literature such as reports, policy literature, government documents for any relevant data. The references of all identified studies were searched again to find other relevant studies. Any disagreement between reviewers regarding any text inclusion was solved by the third reviewer (JP) with a proper discussion.

Selection the studies:

Eligibility criteria: The inclusion criteria for selection of studies were framed according to the population, concept, and context (PCC) framework.

Population: Adolescent girls aged 10–19 were included in the scoping review. The studies with adolescent female-specific data were only included.

Concept: The present scoping review used the perceptions, practices, and understanding of teenage pregnancy among adolescent girls. Studies that described the experiences of pregnancy among married and unmarried adolescents were also considered.

Context: The studies published between the year 2000 and 2021 were considered for this scoping review. The context was not limited to urban or rural settings. All the Hospital-based, school-based, college-based, and community-based studies conducted among Indian adolescent girls were included in the review. All type of study designs encompassing a comprehensive range of relevant papers concerning the perceptions, practices, and understanding related to teenage pregnancy among Indian adolescent girls were included in the study. Only articles published in English were considered.

### Exclusion criteria

- Studies that were undertaken prior to the year 2000 and after 2021.
- Studies conducting other than the Indian population.
- Studies including individuals below 10 years and above 19 years.
- Papers with no appropriate original data on perception, practices, understanding and experiences around teenage pregnancy were excluded.
- Adolescent population where male-specific data were provided.

The selection of the relevant studies was undertaken through different stages. Based on the initial screening, the duplicate articles were evaluated and excluded. Further, the titles and abstracts of the articles were screened to identify eligible articles. Then the inclusion process was undertaken by full-text analysis of the selected studies. All the stages of the inclusion and exclusion procedures used for the selection of studies were presented in the flow diagram as prescribed in Preferred Reporting Items for Systemic Review and Meta-analysis Scoping Reviews (PRISMA-ScR) [31] (Additional file 2).

### Data charting process

The data were extracted from the selected articles as per the objectives of the study and presented in an Microsoft Excel spreadsheet. For the initial screening of the studies, we used a predefined and pretested screening tool (Additional file 5). After the screening, the selected studies with the author's name, year of the study, regions, age group, sample size, sampling methods, study designs, methods of data collection, perception, and practices of Indian adolescent girls about teenage pregnancy and past pregnancy experiences of a married and unmarried adolescent girl were listed systematically in the spreadsheet for the future analysis (Additional file 3). The screening as well as charting was interchangeably done by both the researchers. Discrepancies between reviewers were resolved through consultation among the reviewers in the team.

### Summarizing and reporting the results

After the data extraction, the results were summarized by two reviewers (A.P and S.J) together and any disagreement among them was resolved with the consultancy of the third reviewer (J.P) for the further analysis process. Then, the extracted data were categorized into different aspects according to the study’s objectives, which gave a precise analysis to answer the research questions. A thematic content analysis was conducted to define the major thematic areas. The results of the scoping review involving the selected studies were analyzed around the selected thematic areas.

## Results

### Literature characteristics

**Fig. 1:**
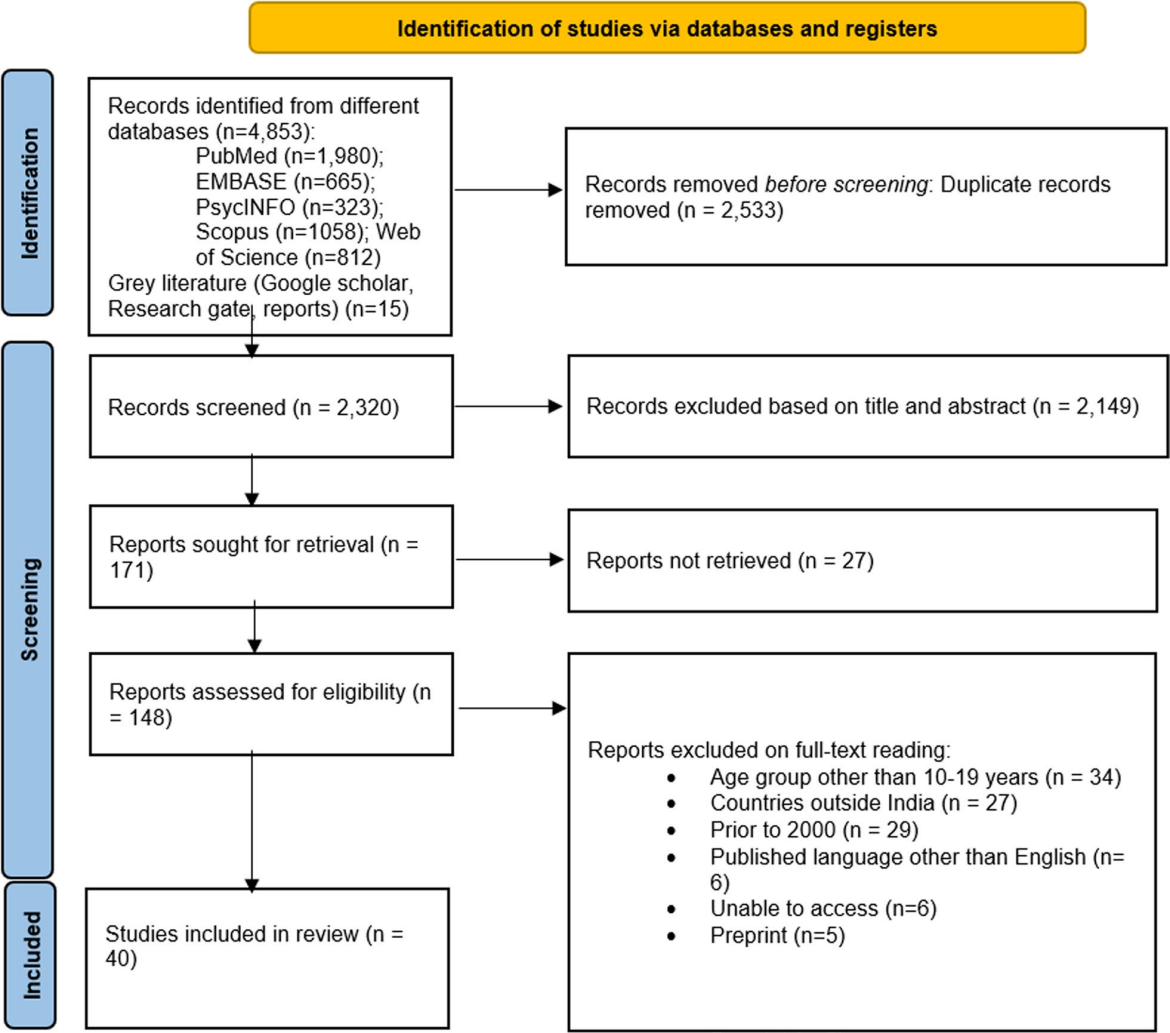
PRISMA flow chart (literature search result)

We explored all the aspects of teenage pregnancy, and the comprehensive search yielded 4853 articles. From the initial investigation, 2533 duplicate articles were excluded. A total of 2149 studies were excluded through the title and abstract screening and 171 papers were selected for full-text screening. After the assessment of 171 full-text articles, 148 articles were excluded based on specific reasons: studies did not include the age group between 10 and 19 (n = 34); outside of India (n = 27); not able to access (n = 6); published before the year 2000 (n = 29); language other than English (n = 7); and preprint (n = 5). The final 40 articles were analysed and discussed in the scoping review paper (Fig. 1).

**Fig. 2:**
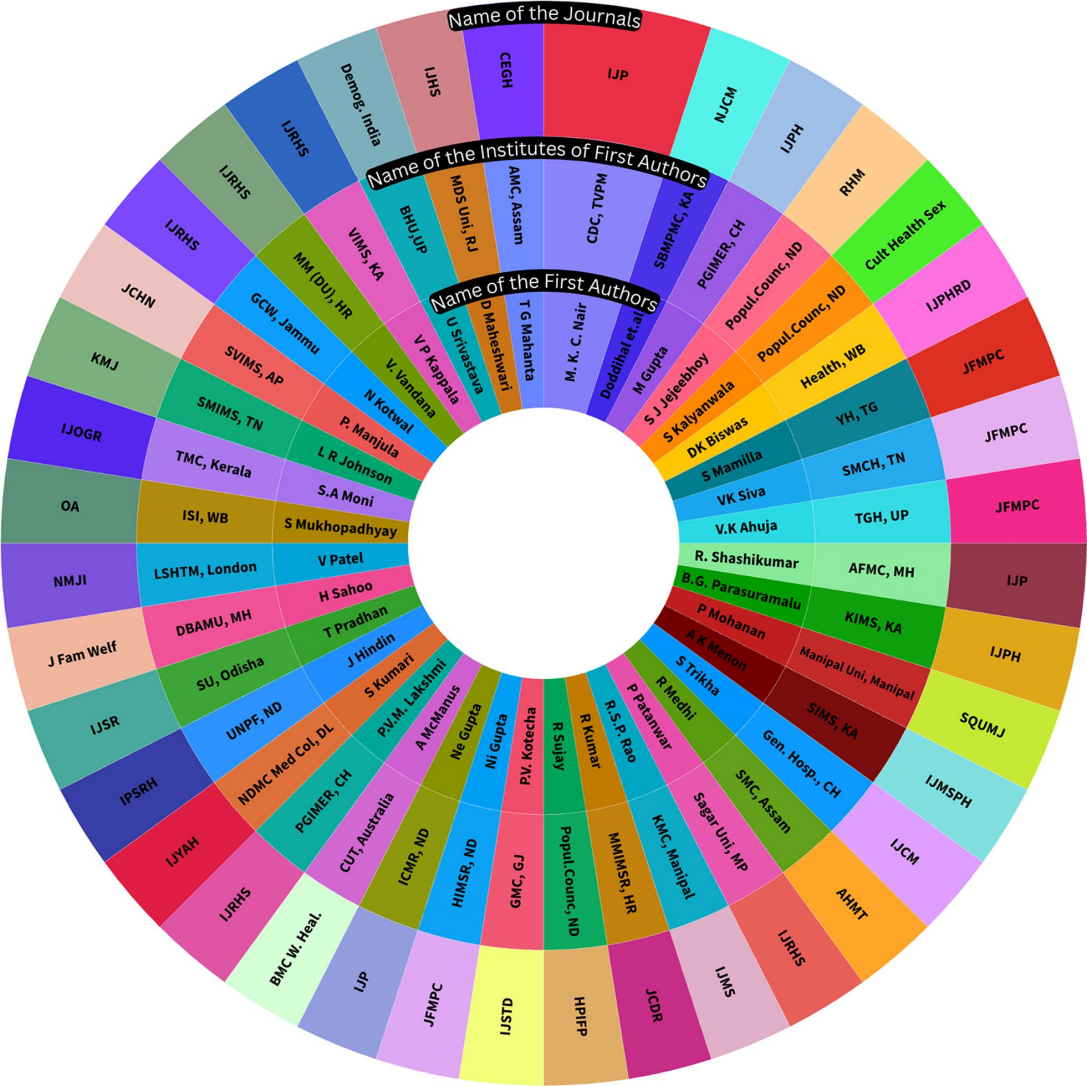
Pattern of the research pursuance, institutional representation, and publishing journals on adolescents in selected studies on teenage pregnancy from India

The bibliographic analysis in Fig. 2 shows the overall pattern of research pursued by individual researchers, institutions where these studies were undertaken, and journals selected for publishing the included studies that focus on teenage pregnancy in the Indian context.

### Overview of studies

A total of forty articles were finally selected and listed as author’s name, journal’s name, institute of 1st author, year of publication, study setting (rural, urban, both rural and urban; Additional file 4), name of the state and union territories in a different region of India, study design, study type, sample size, age group, and method of study, etc.

Selected articles published between 2000 and 2021 were included in the review paper. There was a significant rise in the publication of studies around teenage pregnancy in India in the last decade, focusing on practices, understanding, and perceptions around teenage pregnancy among adolescent girls**.** The included studies were conducted in different regions of India. The highest number of publications were from southern and northern regions, viz. fourteen (n = 14) and eleven (n = 11) respectively. Most studies were quantitative and cross-sectional, i.e., (n = 34). Papers were mainly on the urban population (n = 25), school-based (n = 13), and hospital-based (n = 12) (Table 1).

**Table 1:** Characteristics of the included studies

Year of publication	Number of publications	Percent total publication
2000–2005	3	7.5%
2006–2010	8	20%
2011–2015	14	35%
2016–2020	13	32.5%
2021	2	5%
Type of data		
Qualitative	2	5%
Quantitative	34	85%
Mixed method	4	10%
State		
East	2	5%
West Bengal	1	2.5%
Odisha	1	2.5%
Central	2	5%
Maharashtra	1	2.5%
Chhattisgarh	1	2.5%
West	4	10%
Rajasthan	1	2.5%
Gujarat	2	5%
Goa	1	2.5%
North	11	27.5%
Chandigarh	2	5%
Delhi	4	10%
Jammu and Kashmir	1	2.5%
Haryana	3	7.5%
Uttar Pradesh	1	2.5%
South	14	35%
Tamil Nādu	1	2.5%
Telangana	1	2.5%
Kerala	4	10%
Karnataka	6	15%
Andhra Pradesh	2	5%
Northeast	3	7.5%
Sikkim	1	2.5%
Assam	2	5%
Multi Central	5	12.5%
Bihar, Assam	1	2.50%
Bihar, Jharkhand	1	2.50%
India	2	5%
Rural	5	12.50%
Urban	25	62.50%
Both urban and rural	10	25%
Community-based	9	22.50%
School-based	13	32.50%
College based	6	15%
Hospital based	12	30.00%
Original data	38	95%
Short communication	1	2.5%

### Thematic study analysis

The relevant extracted data from individual studies were analysed from individual studies, and themes were synthesized as follows:

1. **Perception and practices as contributing factors for early pregnancy among adolescent girls**
  The conversations and practices about sexual intercourse are considered immoral and taboo in Indian society. Unsafe sexual intercourse and the perceptions behind it are the major contributing factor to teenage pregnancy among adolescents. It is crucial to study as it explores how adolescents perceive and in what context the practices occur among them. The curiosity about sexual relationships and the unsafe practice of sexual intercourse with wrong perceptions leads to pregnancy in adolescence.
  - **Perceptions among adolescent girls**
    Out of 40 included studies, nine (n = 9) papers discussed the perception of adolescent girls about sexual relationships. A study by Shashikumar et al. (2012) defined girls perceive late-night parties and alcohol consumption are the major factors of sexual relationships among adolescents [32]. Five studies found that girls disapproved of sexual relations before marriage as it is considered unacceptable [33–36]. Also, Nair et al. [35] discussed the perceptions of teenage girls that premarital sex is harmful, unsafe, and dangerous. But three studies (n = 3) examined adolescents’ perception that having premarital sex can be acceptable [32, 34, 37, 38]. Interestingly, one article cited that premarital sex is a cool activity [39], and there was a notion among adolescents that premarital is valid in love relationships if pregnancy can be avoided using contraception [34]. Hindin and Hindin also discussed other vital perceptions among adolescent girls as that sexual relationship is okay in love and to prove love. And sometimes a boy can also force a girl to have sex if he loves her. There was a perception among adolescent girls that one sexual intercourse was enough for pregnancy [36]. One article reported that adolescents perceived early marriage and childbearing as good practices [40]. Only one paper examines the perceptions among girls about the adverse health effects of early pregnancy [53].
  - **Practices among adolescents’ girls**
    Reviewers found ten studies describing practices around teenage pregnancy among adolescent girls. Five articles revealed that adolescents used contraceptives to prevent pregnancy [41–45]. The age of first intercourse was discussed in two papers [32, 41]. Two articles (n = 2) explained that girls experience 1st intercourse between the age of 16 and 18 years [41, 46]. Trikha [44] found the initiation of sexual intercourse among students aged between 14 and 19 years. One article reported that the preference for sexual intercourse among adolescent girls was between the ages of 15 and 19 years [43]. The age gap between sexual contact and sexual intercourse was discussed in one study (n = 1). The average age at first sexual contact was 14 years, whereas, for first intercourse, the average age was 16 years [32].
  - **Practices of contraception and family planning among married adolescents**
    About 12.5% (n = 5) of included studies described the conception and misconception about pregnancy, understanding of contraception and family planning, and use of contraceptives among married adolescent women who have already experienced pregnancy in their lifetime [47–51]. Only one paper highlighted the use of family planning practices among married adolescents. In India, the demand for family planning to keep the age gap between children was higher than limiting families. The met need was more than the unmet need of family planning [51]. Six publications explained contraceptive use and awareness among married adolescents. Two papers discussed that a marginal proportion of adolescent girls used contraception [51, 52]. Contraception knowledge was significantly lower among married teenage women and inadequate education and access to contraceptives resulted in insufficient contraceptive knowledge and use [52]. One article discussed that more than half of married adolescents heard about only condoms, contraceptive pills, and copper-T as contraceptive methods [48]. A significant lack of awareness about family planning among married adolescent girls was highlighted in two studies. In contrast to these studies [47, 50], Only one article reported that most married adolescents were aware of contraception [49].
2. **Experiences and understanding related to pregnancy among adolescents**
  Sexual intercourse with a lack of understanding of contraception, family planning, sex, and abortion can lead to pregnancy. Teenage pregnancy can be avoided with proper knowledge of pregnancy and preventive methods. Pregnancy before marriage is not always socially accepted. When a girl in her adolescence becomes pregnant, abortion is preferred in most cases. Experience of pregnancy and abortion can give an understanding of the teenage pregnancy scenario and its related suffering among adolescent girls.
  - **Understanding of pregnancy and preventive methods among adolescent girls**
    Discussion about understanding can better be explained by the presence of knowledge and awareness among Indian female teenagers. Out of 40 articles, 72% (n = 29) articles discussed the knowledge about contraception and family planning, sex, and abortion. Out of 29 (72%) articles, thirteen articles explained the understanding of sex and pregnancy among adolescent girls [32, 35, 36, 38–41, 45, 53–57]. One paper by Rao et al. examined fundamental knowledge about signs of pregnancy as the missed monthly menstrual cycle. Additionally, two studies described adolescents’ overall understanding of the ovum, sperm, semen, and fertilisation process [38, 55]. Another study by Shashikumar et al. reported that girls understand pregnancy could occur after one sexual intercourse among those who had sexual contact [32]. Teenagers understanding of concepts of physical relations and pregnancy was explained in a study by Patanwar and Sharma [36]. Three articles discussed knowledge and attitude toward the ideal age for childbearing [39, 53, 58].
    Seven articles described the understanding of contraception among adolescent girls between 15 and 19 years [36, 41, 48, 54, 58–61]. Female sterilisation, male sterilisation, Intra Uterine Devices, Copper-T, I-pill, and condoms were the major contraceptives explained by teenage girls [35, 36, 59] but the understanding regarding condoms, oral pills, and emergency contraceptives was described in some of the articles [43, 60–63].
    Knowledge about abortion among adolescent girls was explained in four articles [35, 53, 59, 64]. According to Nair et al., adolescents had knowledge that abortion is a suitable and safe process when a girl becomes pregnant at an early age [35]. Knowledge among girls regarding abortion was explained by one study where it stated that abortion was preferred only in premarital pregnancy and also explained that abortion could be done when the mother and feotus were at risk [64].
    The awareness of contraception among teenage girls was discussed in four papers [43, 44, 62, 65]. Five articles explained the understanding of the use of contraception to prevent unwanted pregnancy [14, 41, 43–45]. Lack of awareness about contraceptive methods and reproductive health was discussed in eight articles [44, 49, 55, 56, 58, 61, 63]. A study by Ahuja et al. (2009) explained consultation after the failure of contraception among adolescents. The doctor was the first preferred choice of consultation in case of a contraceptive failure, while also mothers were also involved in any type of support for adolescents after contraception failure.
    Four studies (n = 4) reported the understanding of family planning among adolescent girls [58, 60, 61, 63]. The understanding of family planning among adolescents was evaluated in terms of avoiding unwanted pregnancy, population control, mothers’ health, and avoiding conception. Three papers stated that family planning was meant to maintain the gap between children [58, 60, 61]. Family planning was necessary, as explained in two papers [60, 61]. Only one article reported awareness of the advantage of family planning among adolescent girls [60].
    The sources by which teenagers gained knowledge about safe sex were described in three papers [14, 33, 41]. Gupta et al. discussed that the internet and partner were the major sources of information about any sexual activity for adolescent girls [41]. Two papers collectively discussed, adolescents gained information about safer sex, mainly from friends, then media, book magazines, internet, and some may consider their parents, health workers, and very least were from school teachers [14, 33].
  - **Experiences of unwanted pregnancy that ended up in abortion among unmarried adolescent girls**
    Among all included manuscripts, three articles discussed the experience of pregnancy and abortion before marriage [17, 18, 44]. Whereas only two qualitative papers discussed in detail the experiences of adolescent girls after pregnancy and the detailed abortion scenarios [17, 18]. A study by Jejeebhoy et al. [17] described that the most consensual relationships lead to pregnancy and resulted in abortion. The reason for abortion was due to pregnancy being the result of forced sexual violence against adolescents as reported in three articles [17, 18, 44]. One article discussed other reasons like pregnancy before marriage and wishing to continue their education were major factors for abortion [17]. One paper reported the psychological trauma among adolescents after the recognition of pregnancy. Girls faced anxiety, fear, and felt guilty being pregnant in the early stage when they were not married [17].
    Studies by Jejeebhoy et al. [17], Kalyanwala et al. [18], and Trikha [44] discussed the reasons for delays in obtaining an abortion after recognition of pregnancy. Adolescent girls recognized their pregnancy after missing two–three menstrual cycles and experiencing vomiting and nausea. After realizing pregnancy, they waited 1 or 2 months before taking any action. The abortion was obtained mostly in the second trimester. Inadequate knowledge about pregnancy and abortion resulted in delays in obtaining abortions among adolescent girls. The studies also described support from partners and families during abortion among unmarried teenage girls. In most cases, the partner was supportive. In fewer cases, when partners were not concerned, they sought help and support from their mother and female friend for abortion [17, 18, 44]. Jejeebhoy et.al [17] reported that in each case, the decision for termination of pregnancy was taken by the parent or partner. Two papers discussed previous attempts to terminate the pregnancy before an abortion. The last effort was conducted mainly by the oral method by approaching the chemist, a nurse, or other uncertified providers [17, 18].
  - **Pregnancy-related understanding and experiences among married adolescents**
    Most pregnant adolescents are married. In Indian society, early marriage and childbearing are shared, and the associated problem related to teenage pregnancy is also high among married adolescents. It is considered that married adolescent girls who experience pregnancy are well-informed and knowledgeable about reproductive and sexual health, but it is not always true. Lack of information about the sexual organ and physiological changes in the opposite sex, pregnancy understanding like what intercourse is, and prevention of unwanted pregnancy lead to unwanted pregnancies and sexual health complications.
    In the present review, four articles discussed the practices of early childbearing and abortion among adolescent girls [47, 50, 66, 67]. Two articles defined the reason for early pregnancy in married adolescent girls. The frequency of teenage pregnancy increased with the increase in cases of early marriage. Family pressure after marriage and the traditional practice of childbearing after marriage [47, 50]. One paper discussed most abortions happened in 1st trimester and few waited till 2nd trimester. Partners, mothers, family, and friends supported adolescents during the abortion [44]. The reason for abortion was not explored in married cases in any of these articles. The studies by Kumari et al. [67] and Biswas et al. [66] discussed the aspect of abortion in married adolescent girls. Most abortions were induced and unsafe. Death cases were also reported due to unsafe abortion in an adolescent girl of 17 years [66]. Unsafe abortion is associated with early pregnancy age, the women’s educational status, and the lower academic qualifications of husbands [67].

## Discussion

This scoping review provides significant insight into the evidence related to perceptions, practices, and understanding of pregnancy among adolescent girls in the Indian context. The results were synthesized from forty articles. In the present scoping review, nearly 20% of papers discussed perceptions about teenage pregnancy among adolescents. Evidence depicted that adolescents mostly perceived sexual relationships as harmful and unsafe before marriage; however, some studies reported sex as accepted and considered cool activity among adolescents. A study by Ezumah et. al. [68] discussed a similar finding that adolescents viewed sexual behaviors as inappropriate for unmarried adolescents. Pre-marital sex was viewed as unacceptable. However, some adolescents perceived pre-marital sex as good [68].

One-fourth of the papers evaluated the practices among adolescent girls. Practices are defined as sexual involvement and use of contraception among adolescents. Older adolescents were more involved in initiating sexual relationships than younger adolescents. More than half of the studies explained the understanding of teenage pregnancy among adolescents. Understanding early pregnancy and sex were the most explored areas. Evidence also explored knowledge about contraceptives, family planning, abortion, and the ideal childbearing age. A similar finding was discussed by Munakampe et al. [69], which also explored knowledge, attitudes, and practices around contraception and abortion among adolescents in low and middle-income countries which described that limited knowledge about sexual and reproductive health among unmarried adolescents was a significant cause of low access to contraception and safe abortion services [69].

The findings showed that very few qualitative studies conducted in India discussed pregnancy scenarios among unmarried adolescents [17, 18]. Reasons for pregnancy, feelings of an unmarried adolescent after confirmation of pregnancy, the reason for delays in obtaining an abortion after recognition of pregnancy, support from partners and family during the abortion, and decision for termination of pregnancy-like aspects were qualitatively explored in the studies involving unmarried adolescents. Previous studies in these aspects reported issues like inadequate knowledge and wrong perceptions about abortion among young girls, delayed recognition of pregnancy, delays in obtaining an abortion, limited understanding of safe abortion services, and fear and shame feeling after pregnancy recognition [21, 70]. The present scoping review explored a paucity of literature in India that addressed the understanding and experience of pregnancy, reproductive health, contraception, use of family planning, early pregnancy, and abortion among married adolescents.

### Major research gaps

There are essential gaps observed concerning the availability of evidence in the present scoping review. Most studies focused on pregnancy around 15–19 years, and only a few addressed sexual behaviours below the age of 15. Very few qualitative studies were conducted around teenage pregnancy in India. The findings showed relatively less work was conducted in the rural setting compared to the urban setting. The perception of rural adolescents was not explored completely. The perception in the included paper was mostly based on the urban setup. The experience part also replicated the story of the urban setup only. There is a major gap in examining rural India in the context of adolescent perceptions, practices, and understanding of teenage pregnancy. The qualitative detailed studies are insufficient to explain the experiences of sexual violence part which is a major factor in pregnancy in India. The experience part was addressed in very few studies. It was found that no studies were available explaining the experience of 1st sexual intercourse and sexual contact without sex. Perception of sexual relations among adolescents was presented in the majority of the studies but perception related to various aspects like type and use of contraceptive methods, how pregnancy occurs, the sign of pregnancy, abortion, etc. are the most neglected areas which need further research. In spite of the fact that teenage pregnancy is a risk due to maternal health complications, but there only one study discussed it very briefly but other papers don’t look at the aspect of the perception of adverse health complications among adolescent girls.

Fasula et al. [71], discussed teenage pregnancy from young men’s perspectives in the United States and identified that men significantly lack knowledge and understanding regarding various aspects of teenage pregnancy, leading to poor attitudes among them toward contraception and utilisation of sexual and reproductive health services. This is a major concern in addressing teenage pregnancy. No studies in India were observed demonstrating teenage pregnancy issues from a male’s point of view [71].

Alongside, gaps were found in practices and understanding about pregnancy among married adolescents like the experiences of early pregnancy, the decision of childbearing after marriage, support of family members and partner during pregnancy, and mental and physical burdens after pregnancy, which need to be discussed for a clear insight upon married adolescent cases. There were few studies that illustrate abortion scenarios like the experiences and trauma of teenage girls during the issues of abortion among Indian adolescent girls. Very few studies explained the pregnancy-related family and social pressure on adolescent girls. The prevailing psychological conditions during post-pregnancy and abortion of teenage girls were presented in very few studies. There is a paucity of literature on the experiences of adolescent girls during the post-abortion period.

The primary study areas of included papers from 2000 to 2021 are shown in Table 2. Study areas like understanding pregnancy, contraception, family planning, and abortion are significant aspects discussed in subsequent years. However, evidence shows that knowledge about the legal age of marriage and childbearing, involvement in premarital sex, and use of contraception among adolescents are explained in relatively few articles. Age in sexual relations, perception about premarital sex, source of information about sex, and consult after the failure of contraception among teenagers are less explored areas as observed in the selected studies in the present review. The study aspects around teenage pregnancy have been more unidirectional in the last two decades. There are limited perspectives explored among adolescents who are sexually active or become pregnant in their adolescence to evaluate the risk behaviour among adolescents.

**Table 2:** The included major study area around perception, practice and understanding of pregnancy among adolescent girls

Year	Major focused study area among adolescent girls	Major focused study area among adolescents who have already experienced pregnancy
2001–2005	• Age at premarital sexual relationship • Perception about premarital • Awareness of contraception • Use of contraception	• Support after pregnancy before marriage • Abortion trimester
2006–2010	• Age at premarital sexual relationship • Perception of premarital sex • Understanding of pregnancy • Understanding of contraception • Understanding of family planning • Involvement in premarital sexual relationship • Sources of information about sex	• Delays in obtaining abortion Support after pregnancy before marriage • Previous unsuccessful attempts to terminate the pregnancy • Decision of termination • Feeling after pregnancy • Reason for early pregnancy • Awareness about family planning
2011–2015	• Age at premarital sexual involvement • Perception of premarital sex • Understanding of pregnancy • Understanding of contraception • Understanding of abortion • Understanding of family planning • Understanding of reproductive health • Understanding of sex • Awareness of contraceptive methods • Knowledge about the ideal age for childbearing • Knowledge about the legal age of marriage for females	• Understanding of contraception • Understanding of family planning • Understanding of sex • Recognition of unintended pregnancy • Abortion trimester • family reaction after pregnancy • Support after pregnancy before marriage
2016–2020	• Perception of premarital sex • Preference for premarital sex • Age at first sexual involvement • Understanding of pregnancy • Understanding of abortion • Understanding of sex • Awareness about contraception • Understanding of family planning • Understanding of Contraception • Use of contraceptive • Understanding regarding early marriage and early pregnancy • Knowledge about the ideal age of childbearing • Consultation after the failure of contraception • Involvement in premarital sexual relationship • Source of knowledge about sex	• Reasons for early pregnancy • Abortion after marriage • Understanding of Contraception • Awareness of contraception • Use contraception
2021	• Perception of premarital sex • Understanding of pregnancy • Understanding of contraception • Use of contraception • Source of knowledge about sex	

Analysis of the research practices around teen pregnancy (Fig. 2) shows that the studies are sporadic, and no research group pursued a consistent focus on the issue. Only one group of authors (Nair et al.) published two papers. Different researchers from three institutes (Population council at New Delhi, Child Development Centre, Medical College at Thiruvananthapuram, and Postgraduate Institute of Medical Education and Research at Chandigarh) were separately involved in seven papers. Most of the studies have been published in public health or allied journals that are reproductive health or adolescence-related journals. This scenario gives a significant research policy dimension highlighting the need for more consistent and focused research on adolescent pregnancy in the Indian context.

We would highlight that none of the papers explored the perspective of a major socio-cultural aspect viz. stigma associated with teen pregnancy among the studied adolescents. Similarly, we observed the roles of religious bodies associated with different Indian religions playing important roles in access to knowledge as well as services/facilities associated with teen pregnancy which is mostly unexplored in research. Various social institutions’ roles in shaping knowledge, perception, and practices of teens around pregnancy have also been largely unexamined in the studies.

The above highlighted gaps show the future scope for potential research and uncovered issues related to teenage pregnancy among Indian adolescents. Although perception, practices, and understanding are crucial issues in teen pregnancy prevention, evidence are not enough to highlight all the issues completely.

### Policy and programmes around teenage pregnancy

Many protective, promotive, and preventive laws, policies, and programs are formulated and implemented by the Government of India for the holistic development of adolescents (Table 3). In the Indian context, as early marriage is the definite reason for early pregnancy. The Ministry of Women and Child Development, Government of India proposed an amendment to increase the minimum age of marriage from 18 to 21 years for girls to prohibit child marriage [72]. As safe abortion is essential in case of adolescent pregnancy, in the same year 2021, another amendment act was passed for medical termination of pregnancy (MPT). In the provision of this act, all women are allowed to seek safe abortion services. The total clinical fee is fully covered by the Government of India [73].

**Table 3:** Acts, policies, and programs around adolescent pregnancy

Protective policies	
The Prohibition of Child Marriage (Amendment) Bill, 2021	• The minimum age at marriage increases from 18 to 21 years for females
The Medical Termination of Pregnancy (Amendment) Act, 2021	• Expands the access to safe and legal abortion services • Increase gestation limit from 20 to 24 weeks for special categories of women, including survivors of rape, victims of incest and other vulnerable women • Extended MTP services irrespective of marital status under the failure of the contraceptive
Promotive policies	
Adolescent Education Programme (AEP)	• This program covers awareness about body image, gender, sexuality, and STIs
Adolescent Reproductive and Sexual Health (ARSH) strategy	• Create preventive, promotive, and curative services for adolescents with public health facilities such as adolescent-friendly health centres (AFHCs) • Enable sexual and reproductive health, Improve knowledge, attitudes, and behaviour, in relation to SRH • Reduce teenage pregnancies • Improve birth preparedness, and complication readiness and provide early parenting support for adolescent parents
Rashtriya Kishor Swasthya Karyakram (RKSK)	• Focused on a holistic health perspective aspect of adolescent reproductive and sexual health like delaying the age of marriage, reducing the incidence of teenage pregnancy, meeting unmet contraception needs, reducing maternal mortality, and reducing STI and HIV cases
Rajiv Gandhi Scheme for Empowerment of Adolescent Girls (RGSEAG)—SABLA	• Enable self-development and empowerment • Promote awareness about health, hygiene, nutrition, Adolescent Reproductive and Sexual Health (ARSH), and family and childcare

As adolescents are the most vulnerable groups and are subjected to limited access to reproductive and sexual knowledge, the Government of India in collaboration with the National AIDS Control Organization (NACO) and the United Nations Children’s Fund (UNICEF), initiated the ‘Adolescent Education Program’ (AEP) in the year 2007 to address this issue. This program covered awareness about body image, gender, sexuality, and STIs; however, due to a lack of national consensus due to the fear that such incorporation of sex education will increase the ‘risky behaviour’ amongst adolescents further [74], the program could not be implemented in several states in India. In 2005, the Ministry of Health and Family Welfare of the Government of India created the Adolescent Reproductive and Sexual Health (ARSH) strategy as a component of the National Rural Health Mission and the Reproductive and Child Health (RCH) programs; this created preventive, promotive, and curative services for adolescents with public health facilities as Adolescent-Friendly Health Centres (AFHCs) [75]. In 2014, the ARSH Strategy was replaced with Rashtriya Kishor Swasthya Karyakram (RKSK). This program focuses on a holistic health perspective of adolescent reproductive and sexual health like delaying the age of marriage, reducing the incidence of teenage pregnancy, meeting unmet contraception needs, reducing maternal mortality, and reducing STI and HIV cases [76]. In 2011, the Ministry of women and child development of India launched a scheme named ‘Rajiv Gandhi Scheme for Empowerment of Adolescent Girls (RGSEAG)—SABLA’ implemented under the Integrated Child Development Service project. The main objective of the program is self-development and empower adolescent girls and promotes awareness about health, hygiene, nutrition, Adolescent Reproductive and Sexual Health (ARSH), and family and childcare [77].

Despite programs and initiatives to reduce teenage pregnancy, teenage pregnancy cases still appear and are rising in certain contexts in India. As the finding suggested and evidence supports, In India adolescent girls mostly having inadequate perception, inaccurate practices, and incomplete understanding of reproductive health which is a major concealed flaw in the increased rate of teenage pregnancy. Comprehensive sex education, providing accurate and age-appropriate information about sexual health, contraception, responsible sexual behaviour, access to contraception, parental involvement, and supportive social service are helpful preventive measures. And the existing policies and programs need timely assessment to focus on gaps and limitations at the grass-root level. Healthcare systems involving adolescent health may be more empathetic with a particular orientation to provide proper medical access to adolescents. Also, in several contexts, reproductive health education, pregnancy, and conception-associated knowledge that are considered taboo for adolescents in Indian society need to be taught more flexibly and in detail in schools. Educators should be trained and oriented to provide information about sexual and reproductive health in schools because they can be the best resource for creating such knowledge and awareness among adolescents. Along with this, the teenage pregnancy issue needs to be viewed through the lens of social and emotional aspects to provide appropriate community-based intervention programs and trained counsellors can provide psychosocial support and proper guidance to the adolescents.

### A framework for scaling-up prevention of teenage pregnancy in India

Addressing the issue of teen pregnancy in India and planning for scalable intervention requires a comprehensive framework that considers multiple factors. In this context, we propose a qualitative framework for scaling-up intervention in India considering the seven key strategies proposed by Cooley et al. [78]. These seven strategies as discussed below provide a comprehensive approach to understanding and analysing the various factors that influence behaviour change and scalability among teenage girls regarding pregnancy.

First, it needs to define the ‘Intervention Recipients**’** or target population, i.e., adolescent girls and/or a particular community; the involvement of various social agencies also cannot be neglected. At this point, it also requires considering the diversity within the target population (e.g., socioeconomic status, education level, regional differences) and its implications for the scalability of intervention. The specific needs, preferences, and characteristics of the intervention recipients need to be evaluated to ensure tailored inputs toward the expected outcome. As the second strategy, it is important to identify the specific ‘intervention content’ targeting teenage pregnancy by considering the relevance, cultural appropriateness, and acceptability in the Indian context. The comprehensiveness and effectiveness of the intervention material in addressing perception, practices, and understanding related to teenage pregnancy also need to be determined. In the third point, ‘Intervention Delivery Agents’ or prospective agencies in the family or community such as individuals or organizations who will be responsible for delivering the intervention need to be identified. Such delivery agencies need to be evaluated regarding their capacity, expertise, and training in implementing the intervention effectively; this is to be followed by assessment of the scalability potential of such chosen delivery agents (e.g., availability, reach, sustainability). Intervention Delivery Channels, fourth, need to be defined for the delivery of intervention for addressing teenage pregnancy (e.g., schools, community centers, digital platforms). The accessibility and feasibility of the chosen delivery channels are further to be assessed, considering factors like infrastructure, technology penetration, and literacy levels; the scalability potential of the selected channels also needs to be evaluated in reaching a larger population. In next, ‘Intervention Implementation’ is another important strategy when the necessary effective strategies and methods for implementing the intervention are finalised. In this strategy, assessing the feasibility, acceptability, and sustainability of the implementation strategies is prioritised by considering the potential barriers and facilitators for successful implementation at scale. No implementation is of any value if not adopted by the target population; so, it needs to evaluate the factors influencing the ‘adoption of the intervention’ among adolescent girls and other target groups as the sixth strategy where the level of engagement, participation, and buy-in from the target population is also assessed parallelly to identify the perceived benefits, motivations, and incentives for adopting the intervention. As the last and seventh strategy as suggested by Cooley et al. [78], we need to understand the ‘Intervention Context’ i.e., broader socio-cultural, economic, and policy context in India regarding teenage pregnancy. It will help in analysing how contextual factors may influence the perception, practices, and understanding related to teenage pregnancy. Most importantly, such a strategy will help in the alignment of the intervention with existing initiatives, policies, and programs. Finally, coming to the point of sustainable funding for scalability in teen pregnancy prevention in India which can be challenging, there are some potential sources of support that could be explored like additional scope for government funding, corporate social responsibility, international aid, Philanthropic foundations, and public–private partnerships. In a vast and diverse country like India, the level of support for change while scaling up the prevention strategy can vary depending on various factors such as cultural norms, political will, stakeholder engagement, and availability of resources. However, by creating a more supportive environment for change, significant progress can be made.

At this point, it may be highlighted that the fit between the intervention and the adopting system is critical for scalability in teen pregnancy prevention. By ensuring that the intervention aligns with the goals, culture, resources, and expertise of the adopting system, it may be possible to increase the likelihood of successful adoption and implementation and ultimately make a significant impact on reducing teen pregnancy rates in the country.

## Strengths and limitations

This is the first scoping review that discussed the evidence around the perceptions, practices, and understanding of pregnancy among adolescent girls and the experiences among married and unmarried teenage girls in the Indian context. The strength lies in the methodology of this scoping review which helps in intensively collecting all evidence about adolescent pregnancy. This study will help to address gaps that can be explored in future research. The study is limited in its nature with respect to the inclusion of studies published in the English language only. The study only utilizes the electronically available data which may have excluded some of the studies published in local and regional journals that are not indexed in major databases. It is also important to note that, due to our exclusion criteria and limited access to potential papers some of the relevant studies may be included.

## Conclusion

Teenage pregnancy is a complex issue that required a multidimensional approach to address. However, evidence around perception, practices, and understanding among adolescents about pregnancy are limited in several perspectives. Scaling up the evidence will give a detailed explanation of the status of existing issues and can give a proper dimension to future policy-level interventions. Focus on research around the identified gaps will support addressing misperception about sexual involvement, pregnancy, conceptions, early sexual initiation, lack of complete understanding about contraception, family planning and abortion that are contributing factors for early pregnancy among adolescent girls in India. It needs to emphasize complete sex education programmes and expanding access to practical and affordable health services to address the consequences of early pregnancy. Addressing the underlying causes of early pregnancy, educating, empowering adolescent girls, and encouraging teenagers to make responsible choices regarding their sexual health are important aspects of the prevention of teenage pregnancy. Also, the present literature review highlighted several limitations in available evidence and along with gaps in research needs serious attention.

### Supplementary Information

**Additional file 1.** List of search Mesh terms for Pubmed database.

**Additional file 2.** PRISMA checklist.

**Additional file 3.** Data extraction sheet. Summary of studies on perceptions, practices, and understanding around teenage pregnancy among adolescent girls in Indiaand experience, and practices of teenage pregnancy among the adolescent who already experienced pregnancy.

**Additional file 4.** List of journals and institutes of 1st authors.

**Additional file 5.** Initial data charting tool.

## Data Availability

Not applicable.

## Abbreviations

UNICEF: United Nations Children’s Fund
UNPFA: United Nations Population Fund
WHO: World Health Organization
NFHS: National Family Health Survey
PRISMA-P: Preferred Reporting Items for Systematic Review and Meta-Analysis Protocols
PRISMA-ScR: Preferred Reporting Items for Systematic Review and Meta-Analysis extension for Scoping Reviews
PCC: Population, concept, and context
MTP: Medical termination of pregnancy
NACO: National AIDS Control Organization
AEP: Adolescent Education Program
RKSK: Rashtriya Kishor Swasthya Karyakram
RGSEAG: Rajiv Gandhi Scheme for Empowerment of Adolescent Girls
