# Correction: Perception, practices, and understanding related to teenage pregnancy among the adolescent girls in India: a scoping review

**DOI:** 10.1186/s12978-024-01790-5

**Published:** 2024-06-05

**Authors:** Arpita Panda, Jayashree Parida, Susangita Jena, Abinash Pradhan, Sanghamitra Pati, Harpreet Kaur, Subhendu Kumar Acharya

**Affiliations:** 1 ICMR-Regional Medical Research Center, NALCO Nagar, Chandrasekharpur, Bhubaneswar, Odisha 751023 India; 2 Division of Epidemiology and Communicable Diseases (ECD-Tribal Health), ICMR Head Quarters, New Delhi, India

**Correction** **:** **Reprod Health 20, 93 (2023)**

**https://doi.org/10.1186/s12978-023-01634-8**

After publication of this article [1], concerns have been raised about the methodology presented by a third party. The expressed concerns were as follows:

• The paper requires elaboration on the research question.

• The paper requires improvements in the methodology: elaborate on reported usage of the Arksey and O'Malley scoping review framework and Joanna Briggs Institute Reviewers’ Manual, and the Systematic Review and Meta-analysis: Extension for Scoping Review (PRISMA-ScR).

• The paper requires explicit inclusion criteria.

• The paper requires refinement of the discussion section.

• The paper requires confirmation on the articles included in the review.

The original article has been updated by the authors. Extensive corrections have been made, Fig. 1 and Additional File 1 have been updated, and an Additional File 5 (Initial data charting tool) has been added.
