# Supplementary material for: Perception, practices, and understanding related to teenage pregnancy among the adolescent girls in India: a scoping review
Source: Reprod Health. 2023 Jun 21;20:93. doi: 10.1186/s12978-023-01634-8 (PMC10283331; doi:10.1186/s12978-023-01634-8)
Supplement: Supplementary file 1 — Additional file 1. List of search Mesh terms for Pubmed database. [file 12978_2023_1634_MOESM1_ESM.docx]

S1 File: Draft of search strategy to be used using PubMed electronic database

| Components | Search items | Results |
| --- | --- | --- |
| Population | | |
| #1 | Pregnanncy: "pregnancy"[MeSH Terms] OR "pregnancy"[All Fields] OR "pregnancies"[All Fields] OR "pregnancy's"[All Fields] | **544,651** |
| #2 | Teenage: "adolescent"[MeSH Terms] OR "adolescent"[All Fields] OR "teenage"[All Fields] OR "teenager"[All Fields] OR "teenagers"[All Fields] OR "teenaged"[All Fields] OR "teenager's"[All Fields] OR "teenages"[All Fields] | **1,353,020** |
| #3 | Adolescent: "adolescences"[All Fields] OR "adolescency"[All Fields] OR "adolescent"[MeSH Terms] OR "adolescent"[All Fields] OR "adolescence"[All Fields] OR "adolescents"[All Fields] OR "adolescent's"[All Fields] | 1,389,779 |
| #4 | Mother: "mother's"[All Fields] OR "mothered"[All Fields] OR "mothers"[MeSH Terms] OR "mothers"[All Fields] OR "mother"[All Fields] OR "mothering"[All Fields] | 190,109 |
| Context | | |
| #5 | India: "india"[MeSH Terms] OR "india"[All Fields] OR "india's"[All Fields] OR "indias"[All Fields] | 595,542 |
| Concept | | |
| #6 | Married: "marriage"[MeSH Terms] OR "marriage"[All Fields] OR "married"[All Fields] OR "marrieds"[All Fields] OR "marries"[All Fields] OR "marry"[All Fields] OR "marrying"[All Fields] | 37,849 |
| #7 | Unmarried: "single person"[MeSH Terms] OR ("single"[All Fields] AND "person"[All Fields]) OR "single person"[All Fields] OR "unmarried"[All Fields] OR "unmarrieds"[All Fields] | 15,141 |
| #8 | **Abortion:** "abort"[All Fields] OR "aborted"[All Fields] OR "aborter"[All Fields] OR "aborters"[All Fields] OR "aborting"[All Fields] OR "abortion's"[All Fields] OR "abortion, induced"[MeSH Terms] OR ("abortion"[All Fields] AND "induced"[All Fields]) OR "induced abortion"[All Fields] OR "abortion"[All Fields] OR "abortions"[All Fields] OR "abortive"[All Fields] OR "abortively"[All Fields] OR "abortives"[All Fields] OR "aborts"[All Fields] | 52,532 |
| #9 | **Knowledge AND Awareness AND Adolescent**  (("knowledge"[MeSH Terms] OR "knowledge"[All Fields] OR "knowledge s"[All Fields] OR "knowledgeability"[All Fields] OR "knowledgeable"[All Fields] OR "knowledgeably"[All Fields] OR "knowledges"[All Fields]) AND ("awareness"[MeSH Terms] OR "awareness"[All Fields] OR "aware"[All Fields] OR "awarenesses"[All Fields]) AND ("adolescences"[All Fields] OR "adolescency"[All Fields] OR "adolescent"[MeSH Terms] OR "adolescent"[All Fields] OR "adolescence"[All Fields] OR "adolescents"[All Fields] OR "adolescent s"[All Fields])) AND (2000:2022[pdat]) | 9,441 |
| #10 | **Practice AND Adolescent**  (("practicability"[All Fields] OR "practicable"[All Fields] OR "practical"[All Fields] OR "practicalities"[All Fields] OR "practicality"[All Fields] OR "practically"[All Fields] OR "practicals"[All Fields] OR "practice"[All Fields] OR "practice s"[All Fields] OR "practiced"[All Fields] OR "practices"[All Fields] OR "practicing"[All Fields]) AND ("adolescences"[All Fields] OR "adolescency"[All Fields] OR "adolescent"[MeSH Terms] OR "adolescent"[All Fields] OR "adolescence"[All Fields] OR "adolescents"[All Fields] OR "adolescent s"[All Fields])) AND (2000:2022[pdat]) | 131,468 |
| #11 | **Experiences AND Pregnancy**  (("experience"[All Fields] OR "experience s"[All Fields] OR "experiences"[All Fields]) AND ("pregnancy"[MeSH Terms] OR "pregnancy"[All Fields] OR "pregnancies"[All Fields] OR "pregnancy s"[All Fields])) AND (2000:2022[pdat]) | 30,208 |
| #12 | #2 AND #1 AND #5  (("adolescent"[MeSH Terms] OR "adolescent"[All Fields] OR "teenage"[All Fields] OR "teenager"[All Fields] OR "teenagers"[All Fields] OR "teenaged"[All Fields] OR "teenager s"[All Fields] OR "teenages"[All Fields]) AND ("pregnancy"[MeSH Terms] OR "pregnancy"[All Fields] OR "pregnancies"[All Fields] OR "pregnancy s"[All Fields]) AND ("india"[MeSH Terms] OR "india"[All Fields] OR "india s"[All Fields] OR "indias"[All Fields])) AND (2000:2022[pdat]) | 1589 |
| #13 | #3 OR #2 AND #1 AND #5  (("adolescences"[All Fields] OR "adolescency"[All Fields] OR "adolescent"[MeSH Terms] OR "adolescent"[All Fields] OR "adolescence"[All Fields] OR "adolescents"[All Fields] OR "adolescent s"[All Fields] OR ("adolescent"[MeSH Terms] OR "adolescent"[All Fields] OR "teenage"[All Fields] OR "teenager"[All Fields] OR "teenagers"[All Fields] OR "teenaged"[All Fields] OR "teenager s"[All Fields] OR "teenages"[All Fields])) AND ("pregnancy"[MeSH Terms] OR "pregnancy"[All Fields] OR "pregnancies"[All Fields] OR "pregnancy s"[All Fields]) AND ("india"[MeSH Terms] OR "india"[All Fields] OR "india s"[All Fields] OR "indias"[All Fields])) AND (2000:2022[pdat]) | 1643 |
| #14 | #7 AND #3 AND #4 AND #5  (("single person"[MeSH Terms] OR ("single"[All Fields] AND "person"[All Fields]) OR "single person"[All Fields] OR "unmarried"[All Fields] OR "unmarrieds"[All Fields]) AND ("adolescences"[All Fields] OR "adolescency"[All Fields] OR "adolescent"[MeSH Terms] OR "adolescent"[All Fields] OR "adolescence"[All Fields] OR "adolescents"[All Fields] OR "adolescent s"[All Fields]) AND ("mother s"[All Fields] OR "mothered"[All Fields] OR "mothers"[MeSH Terms] OR "mothers"[All Fields] OR "mother"[All Fields] OR "mothering"[All Fields]) AND ("india"[MeSH Terms] OR "india"[All Fields] OR "india s"[All Fields] OR "indias"[All Fields])) AND (2000:2022[pdat]) | 18 |
| #15 | #6 AND #3 AND #4 AND #5  (("marriage"[MeSH Terms] OR "marriage"[All Fields] OR "married"[All Fields] OR "marrieds"[All Fields] OR "marries"[All Fields] OR "marry"[All Fields] OR "marrying"[All Fields]) AND ("adolescences"[All Fields] OR "adolescency"[All Fields] OR "adolescent"[MeSH Terms] OR "adolescent"[All Fields] OR "adolescence"[All Fields] OR "adolescents"[All Fields] OR "adolescent s"[All Fields]) AND ("mother s"[All Fields] OR "mothered"[All Fields] OR "mothers"[MeSH Terms] OR "mothers"[All Fields] OR "mother"[All Fields] OR "mothering"[All Fields]) AND ("india"[MeSH Terms] OR "india"[All Fields] OR "india s"[All Fields] OR "indias"[All Fields])) AND (2000:2022[pdat]) | 74 |
| #16 | # 9 AND #1 AND #5  (("knowledge"[MeSH Terms] OR "knowledge"[All Fields] OR "knowledge s"[All Fields] OR "knowledgeability"[All Fields] OR "knowledgeable"[All Fields] OR "knowledgeably"[All Fields] OR "knowledges"[All Fields]) AND ("percept"[All Fields] OR "perceptibility"[All Fields] OR "perceptible"[All Fields] OR "perception"[MeSH Terms] OR "perception"[All Fields] OR "perceptions"[All Fields] OR "perceptional"[All Fields] OR "perceptive"[All Fields] OR "perceptiveness"[All Fields] OR "percepts"[All Fields]) AND ("adolescences"[All Fields] OR "adolescency"[All Fields] OR "adolescent"[MeSH Terms] OR "adolescent"[All Fields] OR "adolescence"[All Fields] OR "adolescents"[All Fields] OR "adolescent s"[All Fields]) AND ("pregnancy"[MeSH Terms] OR "pregnancy"[All Fields] OR "pregnancies"[All Fields] OR "pregnancy s"[All Fields]) AND ("india"[MeSH Terms] OR "india"[All Fields] OR "india s"[All Fields] OR "indias"[All Fields])) AND (2000:2022[pdat]) | 17 |
| #17 | #9 AND #6 AND #8 AND #5  ("awareness"[MeSH Terms] OR "awareness"[All Fields] OR "aware"[All Fields] OR "awarenesses"[All Fields]) AND ("marriage"[MeSH Terms] OR "marriage"[All Fields] OR "married"[All Fields] OR "marrieds"[All Fields] OR "marries"[All Fields] OR "marry"[All Fields] OR "marrying"[All Fields]) AND ("adolescences"[All Fields] OR "adolescency"[All Fields] OR "adolescent"[MeSH Terms] OR "adolescent"[All Fields] OR "adolescence"[All Fields] OR "adolescents"[All Fields] OR "adolescent s"[All Fields]) AND ("abort"[All Fields] OR "aborted"[All Fields] OR "aborter"[All Fields] OR "aborters"[All Fields] OR "aborting"[All Fields] OR "abortion s"[All Fields] OR "abortion, induced"[MeSH Terms] OR ("abortion"[All Fields] AND "induced"[All Fields]) OR "induced abortion"[All Fields] OR "abortion"[All Fields] OR "abortions"[All Fields] OR "abortive"[All Fields] OR "abortively"[All Fields] OR "abortives"[All Fields] OR "aborts"[All Fields]) AND ("india"[MeSH Terms] OR "india"[All Fields] OR "india s"[All Fields] OR "indias"[All Fields])) AND (2000:2022[pdat]) | 8 |
| #18 | #10 AND #7 AND #1 AND #5  ("experience"[All Fields] OR "experience s"[All Fields] OR "experiences"[All Fields]) AND ("single person"[MeSH Terms] OR ("single"[All Fields] AND "person"[All Fields]) OR "single person"[All Fields] OR "unmarried"[All Fields] OR "unmarrieds"[All Fields]) AND ("gravidity"[MeSH Terms] OR "gravidity"[All Fields] OR "pregnant"[All Fields] OR "pregnants"[All Fields]) AND ("adolescences"[All Fields] OR "adolescency"[All Fields] OR "adolescent"[MeSH Terms] OR "adolescent"[All Fields] OR "adolescence"[All Fields] OR "adolescents"[All Fields] OR "adolescent s"[All Fields]) AND ("india"[MeSH Terms] OR "india"[All Fields] OR "india s"[All Fields] OR "indias"[All Fields]) | 1 |
| #19 | #10 AND #6 AND #1 AND #5  (("practicability"[All Fields] OR "practicable"[All Fields] OR "practical"[All Fields] OR "practicalities"[All Fields] OR "practicality"[All Fields] OR "practically"[All Fields] OR "practicals"[All Fields] OR "practice"[All Fields] OR "practice s"[All Fields] OR "practiced"[All Fields] OR "practices"[All Fields] OR "practicing"[All Fields]) AND ("marriage"[MeSH Terms] OR "marriage"[All Fields] OR "married"[All Fields] OR "marrieds"[All Fields] OR "marries"[All Fields] OR "marry"[All Fields] OR "marrying"[All Fields]) AND ("adolescences"[All Fields] OR "adolescency"[All Fields] OR "adolescent"[MeSH Terms] OR "adolescent"[All Fields] OR "adolescence"[All Fields] OR "adolescents"[All Fields] OR "adolescent s"[All Fields]) AND ("pregnancy"[MeSH Terms] OR "pregnancy"[All Fields] OR "pregnancies"[All Fields] OR "pregnancy s"[All Fields]) AND ("india"[MeSH Terms] OR "india"[All Fields] OR "india s"[All Fields] OR "indias"[All Fields])) AND (2000:2022[pdat]) | 58 |
| #20 | #10 AND #7 AND #1 AND #5  (("practicability"[All Fields] OR "practicable"[All Fields] OR "practical"[All Fields] OR "practicalities"[All Fields] OR "practicality"[All Fields] OR "practically"[All Fields] OR "practicals"[All Fields] OR "practice"[All Fields] OR "practice s"[All Fields] OR "practiced"[All Fields] OR "practices"[All Fields] OR "practicing"[All Fields]) AND ("single person"[MeSH Terms] OR ("single"[All Fields] AND "person"[All Fields]) OR "single person"[All Fields] OR "unmarried"[All Fields] OR "unmarrieds"[All Fields]) AND ("adolescences"[All Fields] OR "adolescency"[All Fields] OR "adolescent"[MeSH Terms] OR "adolescent"[All Fields] OR "adolescence"[All Fields] OR "adolescents"[All Fields] OR "adolescent s"[All Fields]) AND ("pregnancy"[MeSH Terms] OR "pregnancy"[All Fields] OR "pregnancies"[All Fields] OR "pregnancy s"[All Fields]) AND ("india"[MeSH Terms] OR "india"[All Fields] OR "india s"[All Fields] OR "indias"[All Fields])) AND (2000:2022[pdat]) | 8 |

Draft of search strategy to be used using Embase electronic database

| Components | Search items | Results |
| --- | --- | --- |
| Population | | |
| #1 | **Pregnancy** | **1,134,670** |
| #2 | **Teenage** | **13,445** |
| #3 | **Teenage Pregnancy** | 12,541 |
| #4 | **Adolescent** | 2,065,747 |
| #5 | **Mother** | 296,838 |
| Context | | |
| #6 | **India** | 1,416,002 |
| Concept | | |
| #7 | **Married** | 50,570 |
| #8 | **Unmarried** | 11,367 |
| #9 | **Abortion** | 144,003 |
| #10 | **Adolescent and Teenage Pregnancy** | 1 |
| #11 | **Adolescent AND Girl AND Teenage AND Pregnancy** | 0 |
| #12 | **Adolescent AND Teenage AND Pregnancy AND India** | 0 |
| #13 | **Knowledge AND Awareness AND Adolescent AND Girls** | 642 |
| #14 | **Practices AND Adolescent AND Girls** | 1,843 |
| #15 | **Perception AND Adolescent AND Girls** | 2,844 |
| #16 | **Experience AND Adolescent AND Girls** | 5,515 |
| #17 | **Knowledge AND Awareness AND Adolescent AND Teenage AND Pregnancy** | 0 |
| #18 | **Perception AND Adolescent AND Teenage AND Pregnancy AND India** | 0 |
| #19 | **Experience AND Adolescent AND Teenage** | 118 |
| #20 | **Experience AND Adolescent AND Teenage AND Pregnancy AND India** | 4 |
| #21 | **Married AND Adolescent AND Teenage AND Pregnancy AND India** | 10 |
| #22 | **Unmarried AND Adolescent AND Teenage AND Pregnancy AND India** | 954 |
| #23 | **Abortion AND Experience AND Adolescent AND Teenage AND Pregnancy AND India** | 0 |

Draft of search strategy to be used using Web of Science electronic database

| Components | Search items | Results |
| --- | --- | --- |
| Population | | |
| #1 | PREGNANCY | **176416** |
| #2 | TEENAGE | **4795** |
| #3 | MOTHER | 69793 |
| #4 | MARRIED | 5610 |
| #5 | UNMARRIED | 867 |
| #6 | ADOLESCENT | 220650 |
| #7 | ADOLESCENT GIRLS | 6915 |
| #8 | ADOLESCENT PREGNANCY OR TEENAGE PREGNANCY | 2808 |
| Context | | |
| #9 | **India** | 420003 |
| Concept | | |
| #10 | ABORTION | 20618 |
| #11 | TEENAGE PREGNANCY AND INDIA | 0 |
| #12 | ADOLESCENT PREGNANCY AND INDIA | 0 |
| #13 | TEENAGE PREGNANCY OR TEENAGE PREGNANCY AND INDIA | 5 |
| #14 | PERCEPTION AND ADOLESCENT GIRLS | 146 |
| #15 | PERCEPTION AND ADOLESCENT GIRLS AND INDIA | 4 |
| #16 | PRACTICES AND ADOLESCENT GIRLS AND INDIA | 8 |
| #17 | UNDERSTANDING AND ADOLESCENT GIRLS AND INDIA | 0 |
| #18 | KNOWLEDGE AND ADOLESCENT GIRLS AND INDIA | 5 |
| #19 | AWARENESS AND ADOLESCENT GIRLS AND INDIA | 3 |
| #20 | KNOWLEDGE AND AWARENESS AND ADOLESCENT GIRLS AND INDIA | 0 |
| #21 | KNOWLEDGE AND AWARENESS AND TEENAGE PREGNANCY AND INDIA | 0 |
| #22 | EXPERIENCES AND TEENAGE PREGNANCY AND INDIA | 0 |
| #23 | EXPERIENCES AND TEENAGE MOTHER AND INDIA | 0 |
| #24 | ABORTION AND ADOLESCENT GIRLS AND INDIA | 0 |
| #25 | REPRODUCTIVE HEALTH AND ADOLESCENT GIRLS AND INDIA | 8 |
| #26 | REPRODUCTIVE HEALTH AND KNOWLEDGE AND ADOLESCENT GIRLS AND INDIA | 0 |

Draft of search strategy to be used using PscyINFO electronic database

| Components | Search items | Results |
| --- | --- | --- |
| Population | | |
| #1 | PREGNANCY | **691** |
| #2 | TEENAGE | **5169** |
| #3 | MOTHER | 56,018 |
| #4 | MARRIED | 18,217 |
| #5 | UNMARRIED | 3,400 |
| #6 | ADOLESCENT | 313,085 |
| #7 | ADOLESCENT GIRLS | 2,449 |
| #8 | ADOLESCENT PREGNANCY OR TEENAGE PREGNANCY | 11,567 |
| Context | | |
| #9 | **India** | 40,316 |
| Concept | | |
| #10 | ABORTION | 3,865 |
| #11 | TEENAGE PREGNANCY AND INDIA | 14 |
| #12 | ADOLESCENT PREGNANCY AND INDIA | 187 |
| #13 | TEENAGE PREGNANCY OR TEENAGE PREGNANCY AND INDIA | 1,280 |
| #14 | PERCEPTION AND ADOLESCENT GIRLS | 99 |
| #15 | PERCEPTION AND ADOLESCENT GIRLS AND INDIA | 1 |
| #16 | PRACTICES AND ADOLESCENT GIRLS AND INDIA | 0 |
| #17 | UNDERSTANDING AND ADOLESCENT GIRLS AND INDIA | 7 |
| #18 | KNOWLEDGE AND ADOLESCENT GIRLS AND INDIA | 0 |
| #19 | AWARENESS AND ADOLESCENT GIRLS AND INDIA | 0 |
| #20 | KNOWLEDGE AND AWARENESS AND ADOLESCENT GIRLS AND INDIA | 1 |
| #21 | KNOWLEDGE AND AWARENESS AND TEENAGE PREGNANCY AND INDIA | 0 |
| #22 | EXPERIENCES AND TEENAGE PREGNANCY AND INDIA | 0 |
| #23 | EXPERIENCES AND TEENAGE MOTHER AND INDIA | 0 |
| #24 | ABORTION AND ADOLESCENT GIRLS AND INDIA | 0 |
| #25 | REPRODUCTIVE HEALTH AND ADOLESCENT GIRLS AND INDIA | 0 |
| #26 | REPRODUCTIVE HEALTH AND KNOWLEDGE AND ADOLESCENT GIRLS AND INDIA | 0 |

Draft of search strategy to be used using Scopus electronic database

| Components | Search items | Results |
| --- | --- | --- |
| Population | | |
| #1 | Term(s): Pregnancy; Year(s): 2000-2021 | **438150** |
| #2 | Term(s): teenage; Year(s): 2000-2021 | **26694** |
| #3 | Term(s): teenage pregnancy; Year(s): 2000-2021 | **8455** |
| #4 | Term(s): adolescent; Year(s): 2000-2021 | **338150** |
| #5 | Term(s): mother; Year(s): 2000-2021 | **400705** |
| #6 | Term(s): adolscents AND TEENAGE PREGNANCY; Year(s): 2000-2021 | **2** |
| Context | | |
| #9 | Term(s): india; Year(s): 2000-2021 | 798650 |
| Concept | | |
| #10 | Term(s): adolscents AND TEENAGE AND PREGNANCY AND INDIA; Year(s): 2000-2021 | 0 |
| #11 | Term(s): KNOWLEDGE AND AWARENESS AND ADOLESCENT AND INDIA; Year(s): 2000-2021 | 4262 |
| #12 | Term(s): PRACTICE AND ADOLESCENT AND GIRL; Year(s): 2000-2021 | 36824 |
| #13 | Term(s): PERCEPTION AND ADOLESCENT AND GIRL; Year(s): 2000-2021 | 2168 |
| #14 | Term(s): EXPERIENCE AND ADOLESCENT AND GIRL; Year(s): 2000-2021 | 444 |
| #15 | Term(s): KNOWLEDGE AND AWARENESS AND ADOLESCENT AND TEENAGE AND ADOLECENT GIRL; Year(s): 2000-2021 | 1 |
| #16 | Term(s): PERCEPTION AND ADOLESCENT AND TEENAGE AND ADOLECENT GIRL; Year(s): 2000-2021 | 2 |
| #17 | Term(s): EXPERIENCE AND ADOLESCENT AND TEENAGE; Year(s): 2000-2021 | 4 |
| #18 | Term(s): EXPERIENCE AND ADOLESCENT AND TEENAGE AND PREGANCY AND INDIA; Year(s): 2000-2021 | 1 |
| #19 | Term(s): MARRIED AND ADOLESCENT AND TEENAGE AND PREGANCY AND INDIA; Year(s): 2000-2021 | 2 |
| #20 | Term(s): UNMARRIED AND ADOLESCENT AND TEENAGE AND PREGANCY AND INDIA; Year(s): 2000-2021 | 8 |
| #21 | Term(s): ABORTION AND EXPERIENCE AND ADOLESCENT AND TEENAGE AND PREGANCY AND INDIA; Year(s): 2000-2021 | 4 |
