# Supplementary material for: Perception, practices, and understanding related to teenage pregnancy among the adolescent girls in India: a scoping review
Source: Reprod Health. 2023 Jun 21;20:93. doi: 10.1186/s12978-023-01634-8 (PMC10283331; doi:10.1186/s12978-023-01634-8)
Supplement: Supplementary file 3 — Additional file 3. Data extraction sheet. Summary of studies on perceptions, practices, and understanding around teenage pregnancy among adolescent girls in Indiaand experience, and practices of teenage pregnancy among the adolescent who already experienced pregnancy. [file 12978_2023_1634_MOESM3_ESM.docx]

**S3 File: Summary of studies on perception, practices, and understanding of teenage pregnancy among adolescent girls in India (n= 31) and experience, and practices of teenage pregnancy among the adolescent who already experienced pregnancy (n= 10)**

| Author | Study setting | Study location | State | Study design | Study type | Sample size | Age group | Perception and practice of adolescent girls (n=30) |
| --- | --- | --- | --- | --- | --- | --- | --- | --- |
| [**Gupta et.al**](#_Gupta_N,_Anwar) **(2020)** | Urban | Hospital-based | Delhi | Quantitative | Cross-sectional study | 165 | 10-19 | **Knowledge of sex**-42 (25.4%) **Premarital sex**-28 (66.7%) **Prevalence of activity was** 16.9% (28/165) **Source of knowledge about sex**-internet (n = 19, 45%) partner (n = 16, 38%) **Age at 1st intercourse**- 16 years (n = 7, 25%),17 year (n = 13, 46.4%),18 years (n = 5, 17.8%) **Knowledge of contraception**-26 (61.9%) Using the contraceptive method-9 ,barrier method -5, (EC) pill – 4 |
| [**Gupta et.al**](#_Gupta,_M.,_Bhatnagar,) **(2015)** | Both rural and urban | Community-based | Chandigarh | Quantitative | Cross-sectional study | 854 | 10-19 | **Knowledge about contraception**-condom 80(53.6), pill 67(44.9), ECP 64(42.9) received sex education-18 (12) access to ECP-9(6) |
| [**Mamilla and Goundla**](#_Mamilla,_S._and) **(2019)** | Urban | College-based | Telangana | Quantitative | Cross-sectional study | 150 | 14-18 | **Awareness about contraception**-40(32%), pills 13(10.4%), EC 11(8.8%) |
| [**Siva et.al**](#_Siva_VK,_Nesan) **(2021)** | Urban | Hospital-based | Tamil Nādu | Quantitative | Cross-sectional study | 380 | 9th-12^th^ | **Knowledge about pregnancy**-fertilisation of the egg occurs in the uterus- 161 (44.2%) semen contains sperms-79(21.8%) pregnancy is the result of fertilization- 174 (47.8%) **Knowledge about contraception**-is douching, a method of birth control- 48(13.2%) Perception about sex-is it cool to have sex- 10 (2.7%) |
| [**Ahuja et.al**](#_Ahuja_VK,_Patnaik) **(2019)** | Urban | Hospital-based | Andra Pradesh | Quantitative | Cross-sectional  study | 500 | 15-19 | **Preference for premarital sex**- 110 (22%) **Awareness about contraception**- condom 192 (38.40%),oral pills-63 (12.60%),emergency pills-141 (28.20%), no response -104 (20.80%) **Use of contraception**- condom 122 (24.40%)oral pills choice-101 (20.20%),emergency pills 98 (19.60%), no response-179 (35.80%) **consultation after failure of contraception**-(n=268) doctor 202(75.37) mother 52 (19.40) friends 32 (11.94) some other 14 (5.22) |
| [**Shashikumar et.al**](#_Shashikumar_R,_Das) **(2012)** | Urban | School-based | Maharashtra | Quantitative | Cross-sectional study | 586 | 15-18 | **Premarital sexual contact**- 17.18%  **Premarital sex**-03 (1.31%) **Age at 1st sexual contact**- 14.09 yrs. **Age at 1st sexual intercourse**- 16.66 yrs. **Perception about premarital sex**- attending late-night parties is likely to increase indulgence in sex-40.8%  consumption of alcohol to increase indulgence in sex-28.3% |
| [**Mohanan et.al**](#_Mohanan,_P.,_Swain,) **(2013)** | Urban | School-based | Karnataka | Quantitative | Cross-sectional study | 376 | 15-19 | **Premarital sex**-(n=21) |
| [**Menon et.al**](#_Menon_AK,_Kumar) **(2014)** | Urban | Hospital-based | Karnataka | Quantitative | Cross-sectional study | 214 | 15-18 | **Knowledge Right age for childbearing**- 17-19 years 167 (78%), 20-22 years 26 (12%), >22 years 21 (10%) **Unaware of contraception**-9(4%) aware-205 (96%) |
| [**Trikha**](#_Trikha,_S.,_2001.) **(2001)** | Urban | Hospital-based | Haryana | Quantitative | Cross-sectional study | 83 | 10-19 | **Age at 1st sexual intercourse**-14y-2(2.4),15y-19(23.5),16y-14(17.3),17y-12(14.8),18y-21(25.9),19y-13(16.1) **Perception about premarital sex**-friendship lead to sexual relation-42%, incestual relationship-16%,1 case-father **Aware of one contraception**-72% ,not aware of any contraception-28% **Use of contraception**-12 |
| [**Patanwar and Sharma**](#_Patanwar_P,_SharmaKKN.) **(2013)** | Urban | School-based | Chhattisgarh | Quantitative | randomised control trial | 500 | 16-19 | **Knowledge about RH**-(8.8%) **Perception about premarital sex**-disagreed pre-marital sexual relation-98.9%,disapproved premarital sexual relation(37.3%) **Knowledge about contraception**-condoms is the means of safe sex(65.5 %), knew that the pregnancy can be prevent-326 (65.2%), heard about contraceptives- 289 (64.5%), knowledge at least two or more modes of contraception(63.3 %), female sterilization-41(14.1%), male sterilization- 20(6.9%), iud-2(0.6%), cu-t-8(2.7%), tablet- 4(1.3%), condom-26(8.9%), traditional method-5(1.7%), at least 2 method-183(63.3%) **Knowledge about sex and pregnancy-**regarding meaning of physical relation(86.6%) knew the meaning of pregnancy- 313(62.7%) knowledge regarding fertility period among girls- 97 (19.4%) **Knowledge about right age of child bearing**- 393(78.6%) |
| [**Rao et.al**](#_Rao,_R.S.P.,_Lena,) **(2008)** | Rural | College-based | Karnataka | Quantitative | Randomised control trial | 779 | 17-18 | **Knowledge about pregnancy**-the first sign of pregnancy is (missed period)-484 (61.2) fusion of the sperm with the ovum is called (fertilization)- 431 (54.5) **Knowledge about contraception**-oral contraceptives- 277 (35.0) condoms- 146 (18.5) IUD- 88 (11.1) tubectomy- 99 (12.5) vasectomy- 15(1.9) do not know- 422 (53.4) |
| [**Nair et.al**](#_Nair,_M.K.C.,_Thankachi,) **(2012)** | Both rural and urban | School-based | Kerala | Quantitative | Randomised control trial | 560 | 13-17 | **Knowledge about pregnancy**-how conception takes place-IX (n=331) BL-61(18.4) EL-172 (51.9), XI-(n=259) BL 106 (40.9)el202(77.9) no **Knowledge about prevention of pregnancy**-IX -BL246 (63.7) EL- 9 (2.7) XI BL- 174 (67.2) EL 36(13.9) **Knowledge about contraception**-condom for male-IX BL 20 (6.0) EL 115 (34.7) XI BL-45 (17.4) EL-137 (52.9) copper-t for female- IX- BL-26 (7.8) EL 85 (25.7) XI-BL-21 (8.1) EL-149 (57.5) oral pills for female- IX-BL-78 (23.6) EL-161 (48.6) XI- BL60 (23.2) EL- 206 (79.5) **Knowledge about abortion**- IX-BL-25 (7.6) EL-127 (38.4) XI-BL-53 (20.5) EL- 144 (55.6), abortion is safe in early pregnancy- IX-BL-23 (6.9) EL-206 (62.2) XI-BL-34 (13.1) EL-135 (52.1) **Perception about premarital sex**-first sexual act should be only after marriage- IX-BL-103 (31.1) EL-242 (73.1) XI-BL-115 (44.4) EL- 214(82.6), premarital sex is bad/immoral-IX-BL-61 (18.4) EL-149 (45.0) XI-BL-110 (42.5) EL-166 (64.1) premarital sex is unsafe/dangerous-IX-BL-73 (22.1) EL-127 (38.4) XI-BL-45 (17.4) EL-89 (34.4)   (*n= number of sample, BL- Baseline EL- End line) |
| [**Kumar et.al**](#_Kumar,_R.,_Goyal,) **(2017)** | Both rural and urban | School-based | Haryana | Quantitative | Cross-sectional study | 358 | 13-19 | **Knowledge about sex**-sex makes future life easy-164(23.6%), remove myth- 160 (23.0%) need of their age- 119(17.1%) not stated-82(11.0%) |
| [**Sujay**](#_Sujay,_R.,_2009.) **(2009)** | Urban | College-based | Gujarat | Mixed method | Cross-sectional study | 9 | 17-19 | **Age at 1st premarital sexual intercourse**-18 years, 19 years |
| [**Kotecha et.al**](#_Kotecha_PV,_Patel) **(2009)** | Rural | School-based | Gujarat | Quantitative | Cross-sectional study | 340 | 10-19 | **Knowledge about family planning**-yes 115(33.8) no-180(52.9), no response-55(16.1), known family planning methods-condoms- 1 0.2 tubectomy-1 0.2 |
| [**Gupta et.al**](#_Gupta_N,_Anwar) **(2004)** | Both rural and urban | School-based | India | Quantitative | Cross-sectional study | 10-14=2046 15-19=1602 | 10-19 | **Awareness about contraceptive**- 10-14=242 (11.8), 15-19=383 (23.9) |
| [**Mcmanus and Dhar**](#_McManus,_A._and) **(2008)** | Urban | School-based | Delhi | Quantitative | Cross-sectional study | 251 | 17-18 | **Premarital sex**-9%(n = 22) **Perception about premarital sex**-believed that it was not possible for girls to remain a virgin prior to marriage-28% (n = 71), considered contraceptive pill could protect a women from HIV infection - 21% (n = 52) **Sources of information available safer sex** - friends(76%, n = 191), the media (72%, n = 1680), books/magazines (65%, n = 165) internet (52%, n = 132) **Source of knowledge about sex-**mother -24% (n= 61) lady doctors- 9% (n = 23) |
| [**Lakshmi et.al**](#_Lakshmi,_P.V.M.,_Gupta,) **(2007)** | Both rural and urban | School-based | Chandigarh | Quantitative | Cross-sectional  study | 128 | 15-19 | **1st sexual contact**-type of sexual contact kissing-5 (3.9), hugging-2 (1.6), sexual intercourse 1 (0.8), any sexual contact-8 (6.0) **Perception about premarital sex**- girls who did not have any sexual physical contact approved premarital sex-0.8%, girls responded that they would marry even if their partner had premarital sex-0.8% |
| [**Hindin and Hindin**](#_Hindin,_J._and) **(2009)** | Urban | Community-based | Delhi | Quantitative method | Descriptive study | 475 | 15-19 | **Premarital sex**-6% **Perception about sex-**only married people should have a sexual relationship-96%, if a girl and a boy love each other, it is okay to have a sexual relationship-14% sometimes a girl may get involved in a sexual relationship to prove her love-38% sometimes a boy has to force a girl to have sex if he loves her-40%, a boy does not respect a girl who is willing to have a sexual relationship before marriage-76% if reliable contraceptives are used to prevent pregnancy, it is okay to have a sexual relationship-26% |
| [**Pradhan**](#_Pradhan,_T.,_2016.) **(2016)** | Urban | College-based | Odisha | Mixed method | Cross-sectional study | 100 | 15-18 | **Knowledge about pregnancy**-union of sperms and ovum takes place-63(63%)   a boy closely hugs/kisses a girl-1 (1%) do not know-36 (36%) **Knowledge about abortion**- abortion can be done in case when risks involved in carrying pregnancy for the health of the mother and foetus-53% abortion can be done in case of pre-marital pregnancy-37%,female foetus (5%) Perception about premarital sex-girls who did not have any sexual physical contact approved premarital sex-0.8%,girls responded that they would marry even if their partner had premarital sex-0.8%,sexual relationship right-after marriage-76%, only when mature enough-13%, love affair-11% |
| [**Patel and Andrew**](#_Patel,_V._and) **(2001)** | Both rural and urban | School-based | Goa | Mixed method | Cross-sectional study | 381 | 15 | **Premarital sex**-csi* (n= 22) v.other (n=359)-23 v. 2 ,age-adjusted or-17.6,95% ci-4.8-64 p value- <0.001 |
| [**Mukhopadhyay and Mishra**](#_Mukhopadhyay,_S._and) **(2021)** | Both rural and urban | Community-based | Sikkim | Quantitative | Cross-sectional study | 1031 | 15-19 | **Use of contraception**-condom use-(urban- 573) 176(30.72) (rural-458)147(32.10) total-(1031)323(31.33) safer sex-urban-123(21.47), rural-121(26.42), total-244(23.67)  **Source of knowledge about sex**-friends -178 (17.3),parents-5 (0.5),other family members-13 (1.3), health workers -38 (3.7),teachers- 10 (0.1) **Perception about premarital sex**-importance of virginity to an unmarried girl- 689(66.83), sex education should be imparted at home only- 101(9.79) sex education goes against religious beliefs- 128(12.42) **Knowledge about contraception**-contraception should be used to avoid pregnancy- 620(60.14) condoms should be used to avoid sexually transmitted diseases-81(7.86) |
| [**Moni et.al**](#_Moni,_S.A.,_Nair,) **(2013)** | Urban | Hospital-based | Kerala | Quantitative | Case-control study | 181 | 13-19 | **Lack of knowledge about reproductive health**-or-6.48,95% ci-1.91–23.28, p-value-0.0027 |
| [**Johnson et.al**](#_Johnson,_L.R.,_Ravichandran,) **(2014)** | Urban | Hospital-based | Kerala | Quantitative | Cross-sectional study | 100 | 10-19 | **Knowledge about pregnancy**-did not know the symptoms of pregnancy-7% didn’t know how to confirm pregnancy-34%, knew that pregnancy can be avoided-47% **Unaware about contraceptive methods**-60% |
| [**Manjula et.al**](#_Manjula,_P.,_Sreelatha,) **(2016)** | Urban | College-based | Andhra Pradesh | Quantitative | Cross-sectional study | 100 | 15-19 | **Knowledge about pregnancy**- inadequate- 0-27% -25%, moderate 28%-40% - 41%, adequate 41-54% - 34% |
| [**Kotwal et.al**](#_Kotwal,_N.,_Khan,) **(2014)** | Urban | School-based | Jammu and Kashmir | Quantitative | Cross-sectional study | 50 | 14-19 | **Knowledge about pregnancy**-125(88.33), fertile period-34(22.67) **The right age of pregnancy**-127 (84.67) pregnancy prevention- 69 (46) adverse effects of early pregnancy-mother-99(66), child- 118 (78.67) **Knowledge about abortion-**129 (86) legal-121 (80.67), illegal-122(81.33), unsafe abortion-46(30.67), harmful effects- 114(76.5) |
| [**Vandana et.al**](#_Vandana_V,_Simarjeet) **(2017)** | Urban | School-based | Haryana | Quantitative | Cross-sectional study | 200 | 12-18 | **Knowledge regarding early marriage and early pregnancy-** good 21-24- 14 (0.7) average 15-21- 171 (85.5) below average 0-15-15 (7.5) level of attitude regarding early marriage and early pregnancy  moderately favourable 50-70- 105 (52.5) favourable >70- 95(47.50) maximum score=100 minimum score = 20 |
| [**Kappala et.al**](#_Kappala_VP,_Doddaiah) **(2014)** | Both rural and urban | Community-based | Karnataka | Quantitative | Cross-sectional study | 400 | 10-19 | **Knowledge about legal age of marriage for females-** 10-15 years-rural- 6(1.5), urban-6 (1.5), 15-17 years-rural- 7(1.8), urban-10(2.5) ≥‎18‎yrs-rural-190 (47.5), urban-219 (54) do not know-rural-197 (49.3), urban- 165 (41.3)  **Knowledge about right age for child bearing**- 10 - 15 yrs-rural-2 (0.5),urban- 1 (0.3), 15 - 17 yrs-rural-11 (2.8), urban-6 (1.5), ≥‎18‎yrs-rural-77 (19.3), urban- 64 (16) do not know-rural-310 (77.5), urban- 329 (82.3) **Knowledge about family planning-**  1 yr-rural-14 (3.5), urban-12 (3)  2 yr- rural-64 (16), urban-85 (21.3)  3 yr-rural-103 (25.8), urban-80 (20) do not know-rural-219 (54.8), urban-223 (55.8) **Knowledge of contraception**-  yes-rural-91 (22.8), urban-284 (71)  no-rural-309 (77.3), urban-116 (29) **Knowledge on methods of contraception-** abortion-rural-11 (2.8), urban-1 (0.3) condom-rural-35 (8.8), urban-41 (10.3) operation-rural-5 (1.3), urban-4 (1)  o c pills- rural-9 (2.3), urban-8 (2) I pill-rural-29 (7.3), urban-46 (11.5)  do not know-rural-2 (0.5), urban-16 (4) |
| [**Srivastava et.al**](#_Srivastava,_U.,_Singh,) **(2018)** | Urban | School-based | Uttar Pradesh | Quantitative | Cross-sectional study | 1800 | 12-19 | **Knowledge about contraceptives** - condom- 49.4, IUD/copper t- 27.9, oral pills- 28.1, tubectomy- 22.8, vasectomy- 16.1, medical termination of pregnancy (MTP)- 27.5 |
| [**Maheshwari**](#_Maheshwari,_D.,_2019.) **(2019)** | Urban | College-based | Rajasthan | Quantitative | Cross-sectional study | 100 | 17-19 | **Ideal age of child bearing**-(20 – 22)- 26, (23 – 24)- 17, (25 – 26)- 32 ,26 onwards- 7 **knowledge about family planning**  when childbirth does not take place- 4  makes the men and women unable to reproduce- 6, planning the birth of one’s child according to one’s choice, not by chance- 55, the ideal gap between two children(years) (1-2)- 23,(3-4)- 64, 5 onwards- 5 **Advantages of family planning**- a happy life- 10, avoid unwanted pregnancy- 7, population control- 24  **Knowledge about contraception -**condom- 14, oral pills- 17, IUD- 4, other- 11, stage of contraceptive use immediately after marriage- 45, after the birth of the first child- 9, after having the desired number of children- 5, any other- 27 |
| [**Mahanta et.al**](#_Mahanta,_T.G.,_Boruah,) **(2015)** | Rural | Community-based | Assam | Mixed method | Cross-sectional | 528 | 10-19 | **Knowledge about contraception**-- IUCD BL- 41 (7.8) EL- 63 (11.6), oral pills/condom-BL- 120 (22.7) EL-282 (51.9), male sterilization-BL- 3 (0.6) EL-3 (0.6), female sterilization-BL- 16 (3.0) EL- 9 (1.7 **Knowledge about family planning**-IUCD-BL-34 (6.4), EL- 31 (5.7) ,oral pills/condom-BL-102 (19.3) ,EL-254 (46.8), sterilization male- BL- 9 (1.7) EL- 7 (1.3), female sterilization-BL- 25 (4.7) EL- 16 (2.9), don't know-BL- 356 (67.4)EL- 235 (43.3)  **Advantage of family planning**-don't know-BL- 302 (57.2) EL-106 (19.5) the health of mother-BL-126 (23.9) EL-388 (71.5), health of child-BL- 9 (1.7) EL-40 (7.4), economics-BL- 78 (14.8), EL- 9 (1.7) (*BL-Baseline, EL-Endline) |
| **Author** | **Study setting** | **Study location** | **State** | **Study design** | **Study type** | **Sample size** | **Age group** | **Experience of pregnancy among adolescents girls who already experience pregnancy(n=10)** |
| [**Doddihal et.al (2016**](#_Doddihal_C,_Katti)**)** | rural | Hospital-based | Karnataka | quantitative | Cross-sectional study | 144 | 15-19 | **Lack of awareness about contraception -** 30 (20.9) **Reasons for early pregnancy-** Family Pressure 67 (46.5), Tradition 47 (32.6) |
| [**Jejeebhoy et.al**](#_Jejeebhoy,_S.J.,_Kalyanwala,) **(2010)** | Urban | Hospital-based | Bihar, Jharkhand | Qualitative | Cross-sectional study | 26 | 15-19 | **Delays in obtaining abortion support from partner ,family friend Previous unsuccessful attempts to terminate the pregnancy Decision of termination Feeling guilty, scared** |
| [**Kalyanwala et.al**](#_Kalyanwala,_S.,_Jejeebhoy,) **(2012)** | Urban | Hospital-based | Bihar, Jharkhand | Qualitative | Cross-sectional study | 26 | 15-19 | **Recognition of unintended pregnancy Abortion at which trimester Family reaction Support from family and partner** |
| [**Biswas et.al**](#_Kumari_S,_Kishore) **(2016)** | Rural | Community-based | West Bengal | Quantitative | Cross-sectional study | 1116 | 12-19 | **Abortion after marriage-**31.6% (1112) spontaneous-626 (32.23%) induced 590 (31.05%) |
| [**Parasuramalu et.al**](#_Parasuramalu,_B.G.,_Shakila,) **(2010)** | Urban | Hospital-based | Karnataka | Quantitative | Cross-sectional study | 78 | 15-19 | **Reason for early pregnancy-f**amily pressure 45( 57.7)tradition 24 (30.8)  **Lack of awareness family planning-** 32 (41%) **Aware of family planning-**oral pills-25 (86%),condoms 20 (69%), IUD/loops-12 (41% EC pill-4 (14%), injectables among spacing (terminal) methods 3 (10%) , tubectomy (laparoscopic or conventional) 20 (69%),vasectomy (no scalpel or conventional)5 (7.2%) |
| [**Trikha**](#_Trikha,_S.,_2001.) **(2001)** | Urban | Hospital-based | Haryana | Quantitative | Cross-sectional study | 83 | 10-19 | **Support from partner**, family, friend--partner-26, mother-40, relative-22, friend-8 **Abortion on which terminate -**1st trimester-55%,2nd trimester-42% |
| [**Medhi et.al**](#_Medhi,_R.,_Das,) **(2016)** | Urban | Hospital-based | Assam | Quantitative | Case-control study | 165 | 15-19 | **Knowledge of contraception-**13.94 (23) **Use contraception-** 4.24 (7) |
| [**Kumari et.al**](#_Kumari_S,_Kishore) **(2019)** | Urban | Community-based | Delhi | Quantitative | Cross-sectional study | 35 | 15-19 | **Abortion after marriage-induced abortion-**16 (17.6), unsafe abortion- 12 (31.6), safe abortion-4 (9.5) |
| [**Sahoo**](#_Sahoo,_H.,_2011.) **(2011)** | Both rural and urban | Community-based | India | Quantitative | Cross-sectional study |  | 15-19 | **Knowledge of contraception and family planning- Demand of family planning** **Andhra Pradesh** 6.3 (female sterilization-4.9) **use of contraception**-25.0 **Assam** 23.1 (withdrawal method-7.8), **Use of contraception**- 46.6, **Bihar** 4.1 (1.3-rhythm,1.1- condom), **Use of contraception**- 41.4, **Gujarat** 18.6 (8.2-rhythm,4.6-condom), **Use of contraception**- 42.5, **Haryana** 12.4 (4.6-condom,3.6-rhythm), **Use of contraception**- 35.9, **Himachal Pradesh** 12.4 (9.9-condom), **Use of contraception**- 39.1, **Karnataka** 6.9 (4.5-female sterilization), **Use of contraception**-39.7, **Kerala** 13.8 (5.0-condom,5.0-withdrawal), **Use of contraception**- 43.3, **Madhya Pradesh** 8.2 (3.5-condom), **Use of contraception-**35.5, **Maharashtra** 7.5 (2.5-female sterilization), **Use of contraception**- 31.2, **Odisha** 9.6 (3.5-pill), **Use of contraception**- 32.8, **Punjab** 14.6 (8.4-condom) **Use of contraception**-31.3, **Rajasthan** 11.9(7.1-condom), **Use of contraception**- 35.4 **Tamilnadu** 6.9 (2.8-female sterilization), **Use of contraception**-31.4 **Uttar Pradesh** 10.7(5.3-rhythm), **Use of contraception**-45.9 **West Bengal** 38.0 (12.8-pill,4.4-condom, 12.4- rhythm, 5.9-withdrawal), **Use of contraception**-58.3 **India 11.4 (2.3-pill, 3.0-condom, 1.2-female sterilization, 3.2-rhythm,1.4- withdrawal), use of contraception 39.7** |
| [**Nair et.al**](#_Nair,_M.K.C.,_Thankachi,) **(2013)** | Both rural and urban | Community-based | Kerala | Quantitative | Cross-sectional study | 24 | 10-19 | **Knowledge about sex-**  Hymen can rupture without sexual encounter-19 Size of penis and performance- no relationship- 15 The legal age at marriage 18 (f) and 21 (m) -24 ideal age for conceiving 20–30 y- 11 Pregnancy can occur in first unprotected sex- 20 Adolescent pregnancy is harmful to mother and child- 21 Pregnancy will not occur through kissing- 21 Condom prevents pregnancy and HIV/aids- 21 MTP is legally permitted in our country- 4, who is responsible for infertility? (both)- 22  Knowledge about contraception-condom- 22  Oral contraceptive pills- 22 Safe period- 9  Emergency contraceptives- 2  Copper t- 14 |
