# Supplementary material for: Perception, practices, and understanding related to teenage pregnancy among the adolescent girls in India: a scoping review
Source: Reprod Health. 2023 Jun 21;20:93. doi: 10.1186/s12978-023-01634-8 (PMC10283331; doi:10.1186/s12978-023-01634-8)
Supplement: Supplementary file 4 — Additional file 4. List of journals and institutes of 1st authors. [file 12978_2023_1634_MOESM4_ESM.docx]

| **List of Journals** | |
| --- | --- |
| NJCM | National Journal of Community medicine |
| IJPH | Indian Journal of Public Health |
| RHM | Reproductive Health Matters |
| Cult Health Sex | Culture Health Sexuality |
| IJPHRD | Indian Journal of Public Health Research & Development |
| JFMPC | Journal of Family Medicine and Primary Care |
| IJP | Indian Journal of Psychiatry |
| SQUMJ | Sultan Qaboos University Medical Journal |
| IJMSPH | International Journal of Medical Science and Public Health |
| IJCM | Indian Journal of Community Medicine |
| AHMT | Adolescent Health, Medicine and Therapeutics |
| IJRHS | International Journal of Research in Health Sciences |
| IJMS | Indian Journal of Medical Sciences |
| JCDR | Journal of Clinical Diagnostic Research |
| IJP | Indian Journal of Paediatrics |
| HPIFP | Health and Population Innovation Fellowship Programme |
| IJSTD | Indian Journal of Sexually Transmitted Diseases |
| BMC W. Heal. | BMC Women's Health |
| IJYAH | Indian Journal of Youth and Adolescent Health |
| IPSRH | International Perspectives on Sexual and Reproductive Health |
| IJSR | International Journal of Science and Research |
| J Fam Welf | Journal of Family Welfare |
| NMJI | The National Medical Journal of India |
| OA | The Oriental Anthropologist |
| IJOGR | Indian Journal of Obstetrics and Gynaecology Research |
| KMJ | Kerala Medical Journal |
| JCHN | Journal of Community Health Nursing |
| IJRHS | International Journal of Research Health Science |
| Demog. India | Demography India |
| IJHS | International Journal of Home Science |
| CEGH | Clinical Epidemiology and Global Health |

**SF 4: List of journals and Institutes of 1^st^ Authors**

| **List of Institute** | |
| --- | --- |
| SBMPMC, KA | Shri B M Patil Medical College, Vijayapura, Karnataka |
| PGIMER, CH | Postgraduate Institute of Medical Education and Research, Chandigarh |
| Popul.Counc, ND | Population Council, New Delhi |
| Health, WB | Health, Kolkata |
| YH, TN | Yashodha Hospital, Hyderabad, Telangana |
| SMCH, TN | Saveetha Medical College and Hospital, Chennai, Tamilnadu |
| TGH, UP | Tema General Hospital, Tema, Uttar Pradesh |
| AFMC, MH | Armed Forces Medical College, Pune, Maharashtra |
| KIMS, KA | Kempegowda Institute of Medical Sciences, Bangalore, Karnataka |
| Manipal Uni, Manipal | Manipal University, Manipal |
| SIMS, KA | Shimoga Institute of Medical Sciences, Shimoga, Karnataka |
| Gen. Hosp., CH | General Hospital, Chandigard |
| SMC, Assam | Silchar Medical College, Ghungoor, Assam |
| Sagar Uni, MP | Dr. Harisingh Gour University, Sagar, Madhya Pradesh |
| KMC, Manipal | Kasturba Medical College, Manipal |
| MMIMSR, HR | Maharishi Markandeshwar Institute of Medical Sciences and Research, Mullana, Haryana |
| CDC, TVPM | Child Development Center, Medical College,  Thiruvananthapuram |
| GMC, GJ | Government Medical College, Vadodara, Gujurat |
| HIMSR, ND | Hamdard Institute of Medical Sciences & Research, New Delhi |
| ICMR, ND | Indian Council of Medical Research, New Delhi |
| CUT, Australia | Curtin University of Technology, Australia |
| NDMC Med Col, Delhi | North Delhi Municipal Committee Medical College, Delhi |
| UNPF, ND | United Nations Population Fund, New Delhi |
| SU, Odisha | Sambalpur University, Odisha |
| DBAMU, MH | Dr. Babasaheb Ambedkar Marathwada University, Aurangabad, Maharashtra |
| LSHTM, London | The London School of Hygiene & Tropical Medicine, London |
| ISI, WB | Indian Statistical Institute, Kolkata |
| TMC, Kerala | Travancore Medical College, Kollam, Kerala |
| SMIMS, TN | Sree Mookambika Institute of Medical Sciences, Kulasekharam, Tamilnadu |
| SVIMS, AP | Sri Venkateswara Institute of Medical Sciences, Tirupati, Andra Pradesh |
| GCW, Jammu | Govt. College for Women, Jammu |
| MM (DU), HR | M.M. College of Nursing, Mullana, Haryana |
| VIMS, KA | Vijayanagar Institute of Medical Sciences, Bellary, Karnataka |
| BHU, UP | Banaras Hindu University, Varanasi, Uttar Pradesh |
| MDS Uni, RJ | Maharshi Dayanand Saraswati University, Ajmer, Rajasthan |
| AMC, Assam | Assam Medical College, Dibrugarh |
