# Supplementary material for: Perception, practices, and understanding related to teenage pregnancy among the adolescent girls in India: a scoping review
Source: Reprod Health. 2023 Jun 21;20:93. doi: 10.1186/s12978-023-01634-8 (PMC10283331; doi:10.1186/s12978-023-01634-8)
Supplement: Supplementary file 5 — Additional file 5. Initial data charting tool. [file 12978_2023_1634_MOESM5_ESM.docx]

**Additional file: Initial data charting tool**

1. If the citation fulfils the criteria to be identified and characterised as the existing literature/ evidence as per the broad topic of the scoping review?

- Yes, a primary evidence
- No, other evidence eligible as per the criteria
- No, does not fulfil the selection criteria
- No, none of the above
- Cannot tell

1. Is the language of communication English?

- Yes, in English
- No
- Cannot tell

1. Is the citation associated with Indian context?

- Yes, reports India specific
- No
- Cannot tell

Decision making process:

- If the language of communication of the article is other than English, the articles were excluded.
- If the citation does not report Indian context, then it was excluded.
- If the reviewer’s answer was ‘Yes’ to question 1,2 and 3, the article was included for further screening and appraisal and for ‘No’/ No, none of above’ the article was excluded. This details were incorporated in the electronic charting sheet.
- If the reviewer’s initial response is ‘Cannot Tell’ for any of the three questions the full article was retrieved for further final decision. ‘Reviewers selected the “Cannot tell” option if the article may be relevant. Full article was retrieved for all such articles identified as “Cannot Tell” response.
